# Physiology, Heavy Metal Resistance, and Genome Analysis of Two *Cupriavidus gilardii* Strains Isolated from the Naica Mine (Mexico)

**DOI:** 10.3390/microorganisms13040809

**Published:** 2025-04-02

**Authors:** Antonio González-Sánchez, Luis Lozano-Aguirre, Guadalupe Jiménez-Flores, Mariana López-Sámano, Alejandro García-de Los Santos, Miguel A. Cevallos, Sylvie Le Borgne

**Affiliations:** 1 Departamento de Procesos y Tecnología, Universidad Autónoma Metropolitana-Unidad Cuajimalpa, Ciudad de México 05348, Mexico; antonio.gs009@gmail.com; 2 Unidad de Análisis Bioinformáticos, Centro de Ciencias Genómicas, Universidad Nacional Autónoma de México, Cuernavaca 62210, Mexico; llozano@ccg.unam.mx; 3 Laboratorio Clínico, Área de Microbiología, Hospital Regional Instituto de Seguridad y Servicios Sociales de los Trabajadores del Estado, Puebla 72570, Mexico; lupitajf@live.com.mx; 4 Programa de Ingeniería Genética, Centro de Ciencias Genómicas, Universidad Nacional Autonoma de México, Cuernavaca 62210, Mexico; mariana.luna.sol17@gmail.com (M.L.-S.); alex@ccg.unam.mx (A.G.-d.L.S.); 5 Programa de Genómica Evolutiva, Centro de Ciencias Genómicas, Universidad Nacional Autónoma de México, Cuernavaca 62210, Mexico; mac@ccg.unam.mx

**Keywords:** Naica mine, *Cupriavidus gilardii*, complete genome, heavy metal resistance

## Abstract

Here, we report the characterization of two *Cupriavidus* strains, NOV2-1 and OV2-1, isolated from an iron-oxide deposit in an underground tunnel of the Naica mine in Mexico. This unique biotope, characterized by its high temperature (≈50 °C) and the presence of heavy metals, is no longer available for sampling at this time. The genomes of NOV2-1 and OV2-1 comprised two replicons: a chromosome of 3.58 and 3.53 Mb, respectively, and a chromid of 2.1 Mb in both strains. No plasmids were found. The average nucleotide identity and the core genome phylogeny showed that NOV2-1 and OV2-1 belonged to the *Cupriavidus gilardii* species. NOV2-1 and OV2-1 grew up to 48 °C, with an optimal temperature of 42 °C. Discrete differences were observed between *C. gilardii* CCUG38401^T^, NOV2-1, and OV2-1 in the biochemical tests. NOV2-1 and OV2-1 presented resistance to zinc, lead, copper, cadmium, nickel, and cobalt. Several complete and incomplete gene clusters related to the resistance to these heavy metals (*ars*, *czc*, *cop* 1, *sil*-*cop* 2, *cup*, *mmf*, and *mer*) were detected in the genome of these strains. Although further studies are needed to determine the origin and role of the detected gene clusters, it is suggested that the *czc* system may have been mobilized by horizontal gene transfer. This study expands the extreme biotopes where *Cupriavidus* strains can be retrieved.

## 1. Introduction

The Naica mine, located in the state of Chihuahua in the northwestern part of Mexico, is globally renowned for its extraordinary geological formations, particularly the Cave of the Crystals, which harbors the world’s largest gypsum crystals [1]. In addition to being a natural geological wonder, Naica was one of the most important lead and zinc mines in Mexico before it closed its operations for an indefinite time in 2015 due to a flood [2]. At present, the mine and caves are inaccessible. Naica’s water originates from magmatic bodies as well as meteoric infiltrations, and the water that springs in the mine galleries has a temperature between 50 and 60 °C and an almost neutral pH [3]. This water has been classified as calcium–sulfate type and has a low NaCl content and significant amounts of metals, mainly zinc (199.3 μg/L) and lead (78.9 μg/L) [4]. Additionally, high concentrations of arsenic (up to 156.54 ppb) have been detected in waters of the Meoqui–Delicias aquifer, which, given its geographical positioning, is a recharge source of Naica’s water [5].

Since the discovery of the Cave of the Crystals in 2000, research conducted on Naica and its caves has predominantly been centered on the genesis of the crystals and other mineralogical, microclimatological, and paleoenvironmental aspects [1,3,6]. Although it has been suggested that microorganisms present in the Naica thermal aquifer might have participated in crystal genesis [7], only three microbiological studies on Naica have been reported in the literature [8,9,10]. This singular subsurface biotope, combining hot conditions and the presence of heavy metal, is of particular interest both for basic studies on microbial adaptation to extreme environments and for potential environmental applications of the microorganisms residing there.

To predict the type of microorganisms that might have participated in the formation of the giant crystals, [10] studied the microbial diversity in subsurface hydrothermal water springing at 60 °C in the deepest mine galleries (−700/760 m) by cloning and sequencing 16S rRNA amplicons obtained by nested PCR. Microorganisms belonging to the Thaumarchaeota, Euryarchaeota, Betaproteobacteria, Candidate Division OP3 bacteria, Firmicutes, and Alphaproteobacteria were detected in this study. According to these authors, all the retrieved OTUs were autochthonous except a Betaproteobacteria related to a *Delftia* strain from a wastewater plant and *Alcaligenes denitrificans*, an opportunistic pathogen. On their part, [9] have isolated Actinobacteria from the crystals and walls of the cave of the Crystals. The phenotypic tests and 16S rRNA gene sequencing data separated these isolates into two subgroups closely related to the *Prauserella* genus (*Pseudonocardiaceae* family). Finally, we studied the bacteria associated with minerals and hot water springs from caves and tunnels of the Naica mine using culture-dependent and culture-independent (DGGE) approaches. This study revealed the presence of Firmicutes, Alphaproteobacteria, Betaproteobacteria, and Gammaproteobacteria in gypsum crystals, iron oxide crusts, and hot springs [8]. These bacteria were likely autochthonous with some allochtonous components due to human intervention. Isolated bacteria included *Bacillus*, *Brevibacillus*, *Paenibacillus*, *Schlegelella*, *Cupriavidus*, *Pseudoxanthomonas*, and *Lysobacter*.

The study of microorganisms previously isolated in Naica is an opportunity to gather more information on how bacteria adapt to multi-extreme environments. Here, we report the physiological and genomic characterization of two *Cupriavidus gilardii* strains, NOV2-1 and OV2-1, previously isolated from an iron oxide crust within the Naica mine by our group [8,11]. We show that these strains exhibit both temperature and metal tolerance. The genus *Cupriavidus*, “lover of copper” belongs to the Betaproteobacteria class, family Burkholderiaceae and comprises a group of Gram-negative, peritrichously flagellated aerobic rods with chemoheterotrophic or chemolithotrophic metabolism, that do not assimilate glucose and can use several amino acids as sole carbon and nitrogen sources [12,13,14]. This genus presently encompasses 20 described species [15], which have been isolated in many places around the world from a variety of natural and anthropogenic environments as soil, water, wastewater, human clinical samples, Agave rhizosphere, Mimosa root nodules, volcanic mudflow, natural asphalt deposit and International Space station [13,16,17,18]. The *Cupriavidus metallidurans* strain CH34, isolated from a metallurgical plant in Belgium in the 70 s, is the model system for mesophilic bacterial HMR [19]. Related strains have been found in different industrial sites and even in clinical samples [16]. Recently, the *C. gilardii* species has also gained attention for its HMR and as a potential indicator of heavy metals contamination in tropical environments [18,20]. The present work expands the habitats where *Cupriavidus* species, such as *C. gilardii* can be retrieved and again highlights the ability of this genus to adapt and survive under harsh conditions as those found in the singular Naica biotope.

## 2. Materials and Methods

### 2.1. Bacterial Strains and Culture Conditions

The *Cupriavidus* reference strains used in this work, *Cupriavidus metallidurans* LMG1195/CH34, *Cupriavidus necator* LMG8453/N-1, *Cupriavidus taiwanensis* LMG19424, and *Cupriavidus gilardii* CCUG38401^T^, were obtained from the ATCC [21,22,23]. The *Cupriavidus* isolates NOV2-1 and OV2-1 had been previously isolated from a semi-soft iron oxide deposit in a tunnel outside the Cueva de las Velas at a depth of −290 m in the Naica mine and a temperature around 44 °C, as described in [8,11]. The strains were routinely cultured in nutrient broth (BD Bioxon, Becton Dickinson, Mexico) under orbital agitation (200 rpm), at 30 °C for *C. metallidurans* CH34, *C. necator* LMG1199, and *C. taiwanensis* LMG19424 or 42 °C for NOV2-1, OV2-1, and *C. gilardii* CCUG38401^T^. The strains were conserved on nutrient broth agar plates at 4 °C for short-term storage, or in glycerol at −80 °C for long-term storage.

### 2.2. DNA Extraction, Genome Sequencing, Assembly and Annotation

The NOV2-1 and OV2-1 genomic DNA was extracted and purified using the Quick-DNA Fungal/Bacterial Miniprep Kit (Zymo Research, Irvine, CA, USA) following the manufacturer’s instructions. The quality and concentration of the DNA were determined using a NanoDrop 2000 spectrophotometer (Thermo Fisher Scientific, Waltham, MA, USA) and further confirmed by agarose gel electrophoresis. Whole-genome sequencing using the PacBio Sequel and Illumina HiSeq 4000 platform was performed at Macrogen (Seoul, Republic of Korea). The Illumina sequencing reads were subjected to quality control with FastQC v0.11.8 [24]. Read trimming was performed with Trim Galore v0.6.1 [25]. Hybrid genome assemblies were performed with SPAdes v3.12.0 [26,27] and Unicycler v0.4.8. The obtained assemblies were then merged and optimized with Metassembler v1.5 [28,29]. Genome completeness was determined using CheckM (v1.2.2) [29]. The final consensus assembly was subjected to gene prediction and functional annotation using Prokka v1.12 [30] and the NCBI Prokaryotic Genome Annotation Pipeline (PGAP) [31] available at “https://www.ncbi.nlm.nih.gov/refseq/annotation_prok/” (accessed on 10 January 2023).

### 2.3. Physiological Characterization

The effect of temperature on the growth of NOV2-1 and OV2-1 was first estimated by spotting overnight liquid cultures on nutrient agar plates and incubating them at 30, 37, 42, 48, and 50 °C for 24 h. The effect of temperature on the maximum growth rate (μ_max_) was then evaluated in nutrient broth at 35, 37, 40, 42, 45, and 48 °C. For this, overnight cultures grown at 42 °C were used to inoculate 50 mL of fresh medium at an initial optical density at 600 nm (OD_600_) of 0.1 in 125 mL Erlenmeyer flasks. The cultures were grown at 200 rpm for 9 h, and samples were taken every hour to measure the OD_600_ using a BioPhotometer Plus (Eppendorf, Hamburg, Germany) instrument. The μ_max_ at each temperature tested was calculated by linear regression of the data during exponential growth. Biochemical analyses were performed by the VITEK 2 automated system according to the manufacturer’s instructions using Gram-negative bacterium identification cards.

### 2.4. Evaluation of Heavy Metals Tolerance

The NOV2-1 and OV2-1 strains and the *Cupriavidus* reference strains were grown overnight in nutrient broth at 42 °C and 30 °C, respectively, with orbital shaking at 200 rpm. The cultures were then washed twice with M9 minimal salts (Sigma-Aldrich, Burlington, MA, USA) 1× and adjusted to an OD_600_ of 0.2 into M9 minimal salts 1×, serially diluted (10^−1^–10^−5^), and spotted (15 μL) onto Tris-buffered LB Lennox agar, with or without metals in square Petri dishes. (The medium was buffered with Tris to maintain the pH neutrality for the metal tolerance experiments). The Tris-buffered LB Lennox agar composition was as follows (for 1 L): tryptone (10 g), yeast extract (5 g), tris-base (6.06 g), and bacteriological agar (15 g). The pH was adjusted to 7.1. Heavy metals stock solutions (CuCl_2_, CoCl_2_ 6H_2_O, CdCl_2_, Pb(N_2_O_6_), ZnCl_2_, and NiCl_2_) were prepared in Milli-Q water and filter-sterilized. These stock solutions were added to the solid medium to achieve final concentrations of 0.5 to 9 mM.

### 2.5. Comparative Genomics Analyses

A total of 39 *Cupriavidus*, 4 *Burkholderia*, and 2 *Ralstonia* complete genomes were downloaded from the NCBI GenBank repository (Table S1). The genome sequences of *C. gilardii* NOV2-1 and OV2-1 can be accessed under the GenBank accession numbers CP083437.1, CP083438.1, CP083735.1, and CP083736.1. The average nucleotide identity (ANI) was evaluated with PYANI v0.2 (average_nucleotide_adeintity.py) [32]. The core genome was obtained with GET_HOMOLOGUES (v11042019) [33]. The orthologous gene clusters produced by GET_HOMOLOGUES were then processed with GET_PHYLOMARKERS (v2.2.5_9May18) [34] to select optimal phylogenetic markers. The phylogenomic reconstruction was performed with RaxML v8.2.10 using the GTR-GAMMA substitution model with 100 bootstrap replicates [35]. The *Burkholderia* and *Ralstonia* genera were used as an outgroup for phylogenetic analysis. The presence of HMR gene clusters in the NOV2-1, OV2-1, and other *Cupriavidus gilardii* strains genomes used for comparison was investigated by using a bidirectional best hit (BBH) approach. Several HMR gene clusters (*ars*, *cup*, *cop 1*, *czc*, *mer*, *mmf*, *cop 2*, *pbr*, *cnr*, and *ncc*) responsive to the metals studied here have been identified in the genome of the multi-metal-resistant model bacterium *C. metallidurans* CH34 [36,37]. The proteins encoded by these gene clusters were submitted to reciprocal pairwise sequence comparisons between the predicted protein-coding genes of NOV2-1 and OV2-1, *C. gilardii* genomes, and *C. metallidurans* CH34 using BLASTp. Proteins were considered reciprocal best hits and likely orthologs when they presented amino acid identities > 80%, query cover > 80%, e-value near 0, and were part of an operon structure. The *pbr*, *cnr*, and *ncc* clusters, found to be absent in NOV2-1 and OV2-1, were not further investigated in other *C. gilardii* genomes.

## 3. Results

### 3.1. General Properties of the NOV2-1 and OV2-1 Genomes

Table 1 presents the general properties of the NOV2-1 and OV2-1 genomes. The total genome sizes of NOV2-1 and OV2-1 were 5.7 and 5.6 Mb, respectively. The estimated genome completeness was 99.89 and 99.66%, with an estimated contamination of 0.26 and 0.23% for NOV2-1 and OV2-1, respectively, indicating high-quality genomes. The GC contents were 67.5% in both strains. The genomes consisted of two chromosomes of 3.6 Mb and 2.1 Mb for NOV2-1 and, 3.5 Mb and 2.1 Mb for OV2-1. A total of 5024 and 5011 putative coding sequences (CDS) were predicted in NOV2-1 and OV2-1, of which 4095 and 4064 were assigned a function, respectively. Twelve ribosomal RNAs (rRNAs) and 59 transfer RNAs (tRNAs) were predicted in both genomes. Of the rRNAs and tRNAs found in both strains, 9 and 52 were in the largest replicon, respectively.

### 3.2. Phylogenetic Characterization

**Figure 1:**
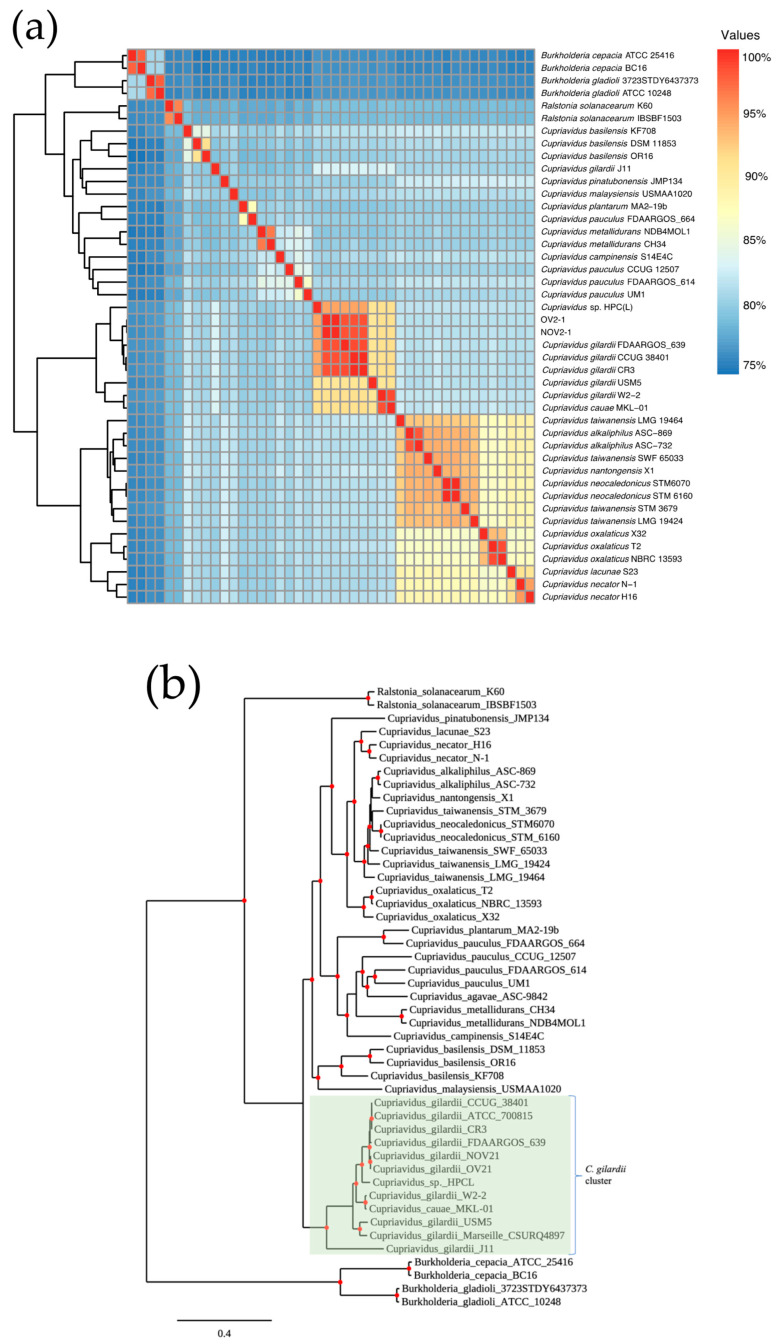
Pairwise genomic ANI values and maximum-likelihood phylogeny of NOV2-1, OV2-1, and other *Cupriavidus* strains. (**a**) Heatmap of ANI. (**b**) Phylogeny generated from 510 concatenated core genes. The *Burkholderia* and *Ralstonia* strains were used as outgroups to root the tree. The scale bar represents the number of substitutions per site.

The genomes of NOV2-1 and OV2-1, 36 other *Cupriavidus* strains from 12 different species, and two unclassified *Cupriavidus* isolates with available whole genome sequences were analyzed by ANI (Figure 1a and Table S2). The ANI values between NOV2-1 and *C. gilardii* CCUG 38401^T^, *C. gilardii* FDAARGOS_639 (reference genome), and *C. gilardii* CR3 were 98.31, 98.45, and 98.48%, respectively. For OV2-1, the ANI values were 98.29, 98.42, and 98.45%, respectively. The pangenome analysis based on 38 *Cupriavidus* genomes and the NOV 2-1 and OV 2-1 genomes allowed for the retrieval of a final set of 510 orthologous genes as phylogenetic markers. The concatenated alignment of these genes was then used to build a phylogenetic tree (Figure 1b) where it can be observed that NOV 2-1 and OV 2-1 grouped with the *C. gilardii* branch. So, according to ANI percentages and the phylogeny, these strains will be referred to as *C. gilardii* NOV2-1 and *C. gilardii* OV2-1. Additionally, the phylogenetic tree also showed that *C. gilardii* J11 branched separately from *C. gilardii* and that *C. taiwanensis* STM 3679 and *C. pauculus* FDAARGOS 664 should be reclassified.

### 3.3. Physiological Characteristics of NOV2-1 and OV2-1

NOV2-1 and OV2-1 are fast-growing, Gram-negative, motile, rod-shaped bacteria that form creamy, slightly domed, and slightly mucoid colonies within 1–2 days when grown on nutrient agar at 42 °C. Table 2 presents a summary of the physiological characteristics of strains NOV2-1 and the reference strains used here. As *C. gilardii* CCUG38401^T^, NOV2-1 and OV2-1 could grow between 30 and 48 °C and no growth was observed at 50 °C while *C. necator* LMG8453 and *C. taiwanensis* LMG19424 only grew up to 37 °C and *C. metallidurans* CH34 up to 30 °C (Figure S1). The two strains had an optimal growth rate between 42 and 45 °C (Figure S2). Growth was better without NaCl in the medium, and growth was reduced by half when 1% (*p*/*v*) of NaCl was added. Concerning pH, both strains exhibited optimal growth at pH 7–8; however, OV2-1 displayed a broader pH tolerance (5.5–9.9) compared to NOV2-1 (7–9.9).

The biochemical tests performed on NOV2-1, OV2-1, and the reference strains (*C. metallidurans* CH34, *C. taiwanensis* LMG19424, and *C. gilardii* CCUG38401^T^) (Table S3) showed that all were positive to L-proline acrylamidase, L-lactate alkalinization, succinate alkalinization, and tyrosine arylamidase. NOV2-1 and OV2-1 were negative for D-glucose and all the other monosaccharides, disaccharides, and sugar alcohols included in the test, as the reference strains. Differences between NOV2-1, OV2-1, and *C. gilardii* CCUG38401^T^ were observed. For example, *C. gilardii* CCUG38401^T^ and all the other reference strains were positive for ELLMAN, while both NOV2-1 and OV2-1 were negative for this test. For L-malate and L-lactate assimilation NOV2-1 and OV2-1 were positive as *C. necator* N-1 and *C. taiwanensis* LMG19424, whereas *C. gilardii* CCUG38401^T^ was negative. OV 2-1, *C. gilardii* CCUG38401^T^ and *C. metallidurans* CH34 gave a positive result for glutamyl arylamidase pNA, whereas NOV2-1 produced a negative result. OV2-1 was positive for citrate (sodium) utilization as *C. necator* N-1, *C. metallidurans* CH34, and *C. taiwanensis* LMG19424, whereas NOV2-1 and *C. gilardii* CCUG38401^T^ were negative.

### 3.4. Tolerance to Heavy Metals

**Figure 2:**
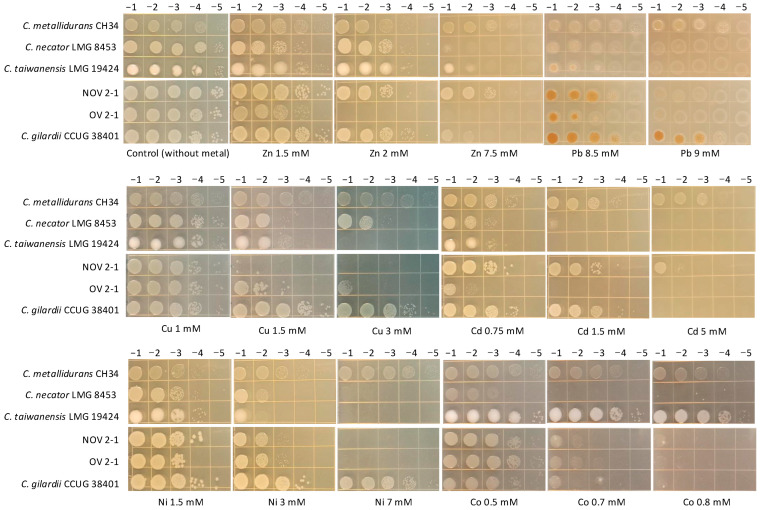
Heavy metals tolerance profiles of NOV 2-1, OV 2-1, and *Cupriavidus* reference strains through agar drop plate method. Serial dilutions (top) of overnight cultures were plated on the Tris-buffered LB Lennox agar amended with increasing concentrations of heavy metals (base of the figures).

As NOV2-1 and OV2-1 were isolated from a mine, their tolerance to heavy metals was tested. The multi-metal-resistant bacterial model *C. metallidurans* CH34 and the other *Cupriavidus* reference strains were included for comparison. NOV2-1, OV2-1, and *C. gilardii* CCUG38401^T^ were tested at 42 °C, and the other reference strains at 30 °C. The results are presented in Figure 2. For all the metals tested, *C. metallidurans* CH34 was the highest tolerant strain, as expected. Differences in metal tolerance were observed between the *C. gilardii* CCUG38401^T^, NOV2-1, and OV2-1.

Concerning Zn, *C. metallidurans* CH34, NOV2-1, *C. taiwanensis* LMG19424, and *C. gilardii* CCUG38401^T^ had the highest tolerance (7.5 mM), followed by *C. necator* LMG8453 (2 mM), and OV2-1 (1.5 mM). Regarding Pb, NOV2-1, OV2-1, *C. taiwanensis* LMG19424, and *C. necator* LMG8453 had similar tolerance levels (8.5 mM). *C. gilardii* CCUG38401^T^ could grow up to 9 mM of this metal as *C. metallidurans* CH34. In the *C. gilardii* strains (NOV2-1, OV2-1, and CCUG38401^T^) and, to some extent, in *C. taiwanensis* LMG19424, bacterial tolerance to Pb was characterized by brown colonies, indicating lead precipitation [38]. For Cu, NOV2-1 did not grow at the lowest concentration tested (1.5 mM), while OV2-1 tolerated up to 3 mM, as *C. metallidurans* CH34 and *C. gilardii* CCUG38401^T^, which both showed full growth at this concentration. *C. taiwanensis* LMG19424 had an intermediate tolerance (1.5 mM of Cu). In the case of Cd, NOV2-1 presented a high tolerance (5 mM), similar to that of *C. metallidurans* CH34, while *C. gilardii* CCUG38401^T^ had a lower tolerance (1.5 mM), followed by *C. necator* LMG 8453, *C. taiwanensis* LMG19424, and OV2-1 (0.75 mM). OV2-1 presented poor growth at this Cd concentration. *C. metallidurans* CH34, and *C. gilardii* CCUG38401^T^ showed full growth up to 7 mM of Ni while the other strains, including NOV2-1 and OV2-1, only tolerated 3 mM of this metal. *C. necator* LMG8453 and *C. taiwanensis* LMG19424 had poor growth at this Ni concentration. Finally, *C. metallidurans* CH34 and *C. taiwanensis* LMG19424 were the most Co tolerant strains (0.8 mM) followed by *C. gilardii* CCUG38401^T^, NOV2-1, OV2-1, and *C. necator* LMG8453 which poorly grew at 0.5 mM of Co.

### 3.5. Genomic Analysis of HMR Gene Clusters

#### 3.5.1. *ars* Cluster

**Figure 3:**
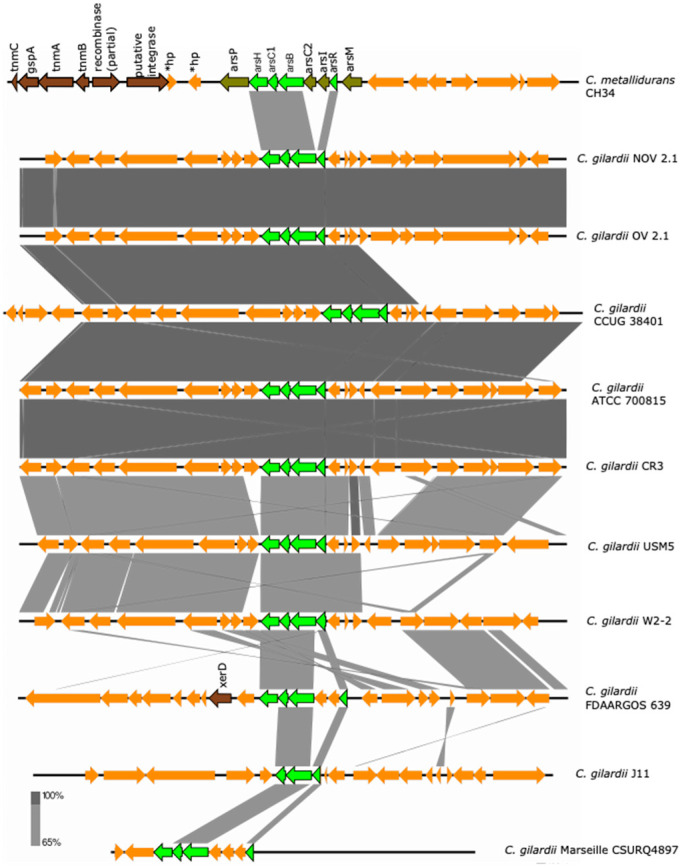
Map showing the genomic organization of the *ars* cluster in *C. metallidurans* CH34, NOV2-1, OV2-1, and other *C. gilardii* strains. The conserved and non-conserved genes of the cluster are colored in neon and olive green, respectively. Orange arrows indicate neighboring genes. The genes colored in brown indicate parts of horizontal DNA transfer-related sequences. *hp means hypothetical protein. The gray scale indicates the levels of synteny.

The *ars* cluster is encoded on chromosome 1 of *C. metallidurans* CH34 and consists of eight genes (*asrP*: MFS permease; *asrH*: NADPH-dependent FMN reductase; *arsC1*: arsenate reductase; *arsB*: arsenite efflux pump; *asrC2*: arsenate reductase; *arsI*: lactoylglutathione lyase; *arsR*: transcriptional regulator; *asrM*: S-adenosyl-L-methionine-dependent methyltransferase) which are maximally activated by the presence of As ions and partially upregulated in the presence of Pb, Zn, Co, Se, Cd and Cu ions [36,39]. An incomplete *ars* cluster was found in chromosome 1 of NOV2-1 and OV2-1 and in all the other *C. gilardii* strains analyzed here (Figure 3).

The *asrH*, *asrC*1, *asrB*, and *asrR* were the conserved genes (except in strain J11 where the *arsH* gene was missing). The *arsRBC1H* proteins of NOV2-1 and OV2-1 displayed 83.2–87.5% amino acids identity to those of *C. metallidurans* CH34 (Table S4). The horizontal DNA transfer-related sequences present in *C. metallidurans* CH34 near the *ars* cluster were not found in *C. gilardii* (Figure S3).

#### 3.5.2. *czc* Cluster

*C. metallidurans* CH34 has two copies of the *czc* cluster. The complete and functional *czc* cluster is encoded on the pMOL30 plasmid and is composed of eleven genes (*czcM*, MgtC-like Mg(II) transport ATPase; *czcN*, isoprenylcysteine carboxyl methyltransferase (regulation); *czcI*, cobalt–zinc–cadmium resistance protein/cation efflux system protein; *czcC*, outer membrane protein/three components cation proton antiporter efflux system; *czcB*, membrane fusion protein/three components cation proton antiporter efflux system; *czcA*, efflux chemiosmotic pump/three components cation proton antiporter efflux system; *czcD*, cation diffusion facilitator; *czcR*, two-component regulator; *czcS*, sensor histidine kinase of the two-component regulatory system; *czcE*, ion binding protein; *czcJ*, hypothetical protein; *czcP*, cation efflux P1-ATPase). This cluster is mainly responsive to Cd, Zn, and Co, although some responses to Pb and Cu have also been detected [36,39].

**Figure 4:**
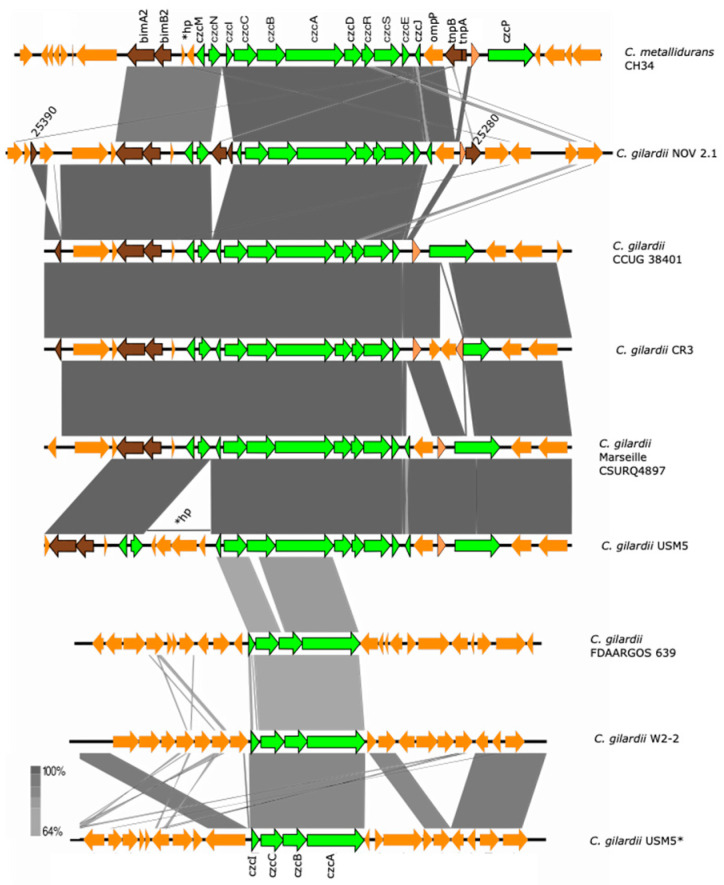
Map showing the genomic organization of the *czc* cluster in *C. metallidurans* CH34, NOV2-1, OV2-1, and other *C. gilardii* strains. The conserved and non-conserved genes of the cluster are colored in neon and olive green, respectively. Orange arrows indicate neighboring genes. The genes colored in brown indicate parts of horizontal DNA transfer-related sequences. *hp means hypothetical protein. The gray scale indicates the levels of synteny.

The complete *czc* cluster was found in *C. gilardii* CCUG38401^T^, CR3, and Marseille CSURQ4897 (Figure 4). An incomplete *czc* cluster missing the *czcP* gene and containing an insertion sequence between *czc*N and *czc*I was found in chromosome 1 of NOV2-1, while this cluster was completely absent in OV2-1. Only the *czcICBA* region was found in *C. gilardii* FDAARGOS 639, W2-2 and USM5. The *czc* proteins of NOV2-1 had 98.6–100% amino acid identity with those of *C. metallidurans* CH34 (Table S5). In NOV2-1, the horizontal DNA transfer-related sequences on the right side of the cluster were not conserved to *C. metallidurans* CH34 (Figure S4).

#### 3.5.3. *cop* Cluster I

The *cop* cluster 1, encoded on chromosome 2 (chromid) of *C. metallidurans* CH34, is constituted by six genes (*copA*, periplasmic multicopper oxidase; *copB*, periplasmic copper resistance protein B; *copC*, periplasmic copper resistance C protein precursor; *copD*, copper resistance protein D transmembrane component; *copR*, DNA-binding response regulator in two-component regulatory system with *CopS*; *copS*, sensory histidine kinase in two-component regulatory system with *CopR*, senses copper ions). This periplasmic detoxification system appears to be responsive only to Cu ions [36].

**Figure 5:**
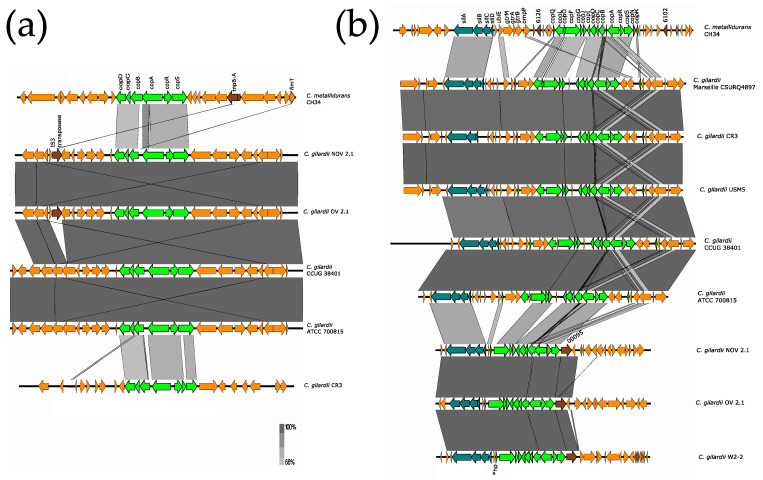
Map showing the genomic organization of the *cop* 1 (**a**) and *sil-cop* 2 (**b**) clusters in *C. metallidurans* CH34, NOV2-1, OV2-1, and other *C. gilardii* strains. The conserved and non-conserved genes of the cluster are colored in neon and olive green, respectively. Orange arrows indicate neighboring genes. The genes colored in brown indicate parts of horizontal DNA transfer-related sequences. *hp means hypothetical protein. The gray scale indicates the levels of synteny.

NOV2-1 and OV2-1 as well as *C. gilardii* CCUG 38401, ATTC 700815 and CR3 were found to harbor the complete *cop* cluster 1 in their chromosome 1 (Figure 5a). This cluster was not detected in the other *C. gilardii* strains included in the study. The *cop* proteins of NOV2-1 and OV2-1 had a low amino acid identity to those of *C. metallidurans* CH34 (from 49.2 to 88.9%) (Table S5). The horizontal DNA transfer sequences at the vicinity of the *cop* 1 cluster were different in *C. gilardii* and in *C. metallidurans* CH34 (Figure S5).

#### 3.5.4. *sil* and *cop* Cluster 2

The *sil* and *cop* cluster 2 are coded contiguously on the pMOL30 plasmid in *C. metallidurans* CH34.

The *sil* cluster consists of four genes (*silA*, proton antiporter metal efflux system; *silB*, proton antiporter metal efflux system; *silC*, outer membrane silver efflux system; *silD*, transmembrane protein). The *sil* cluster is mainly responsive to Ag, and only the *silA* gene is upregulated in the presence of Cu [36].

The *cop* cluster 2 is constituted by the following twenty-one genes: *copW* (hypothetical protein), *copE* (copper resistance protein), *copH* (copper-binding protein), *copQ* (copper protein), *copL* (type II enzyme restriction), *copO* (unknown function), *copF* (P-type ATPase efflux), *copG* (thioredoxin-like protein), *copJ* (putative cytochrome c), *copI* (putative oxido-reductase protein), *copD1* (copper resistance protein D), *copC1* (Cu^2+^-binding protein), *copB1* (outer membrane protein), *copA1* (multi-copper oxidase protein), *copR1* (two-component transcriptional regulator), *copS1* (sensor histidine kinase/two-component regulatory system), *copN* (copper protein), *copK* (Cu^+^ binding protein), *copM* (copper resistance protein), *copT* (putative cytochrome), and *copV* (copper resistance protein). The *cop* cluster 2 responds to Cu, Ag, Cd, Ni, Zn, and Co ions [36].

The *sil-cop* region was present in chromosome 2 (chromid) of NOV2-1 and OV2-1 and in all the *C. gilardii* genomes analyzed except in FDAARGOS 639 and J11 (Figure 5b). Only ten *cop* genes (*copABCDFGIJRS*) were found in *C. gilardii* CR3, and the *silD* gene was absent in this strain. The *sil* and *cop* gene products of NOV2-1 and OV2-1 had 68–89.5% amino acid identities to those of *C. metallidurans* CH34 (Table S5). The horizontal DNA transfer sequences at the vicinity of the *sil*-*cop* 2 cluster were different in *C. gilardii* and in *C. metallidurans* CH34 (Figure S6).

#### 3.5.5. *cup* Cluster

The *cup* cluster is located on chromosome 1 of *C. metallidurans* CH34 and is composed of 3 genes (*cupC*, copper chaperone, heavy metal ion binding; *cupA*, P-type ATPase and *cupR*, DNA-binding transcriptional activator of copper-responsive regulon genes from the MerR family). This cluster exhibits significant upregulation in response to Ag, As, Pb, and Cu ions in *C. metallidurans* CH34 [36,39]. Two extra genes upstream of *cupR*, *betA2* (encoding a choline dehydrogenase) and *pldB* (encoding a lysophospholipase) are also thought to belong to the cluster [36].

**Figure 6:**
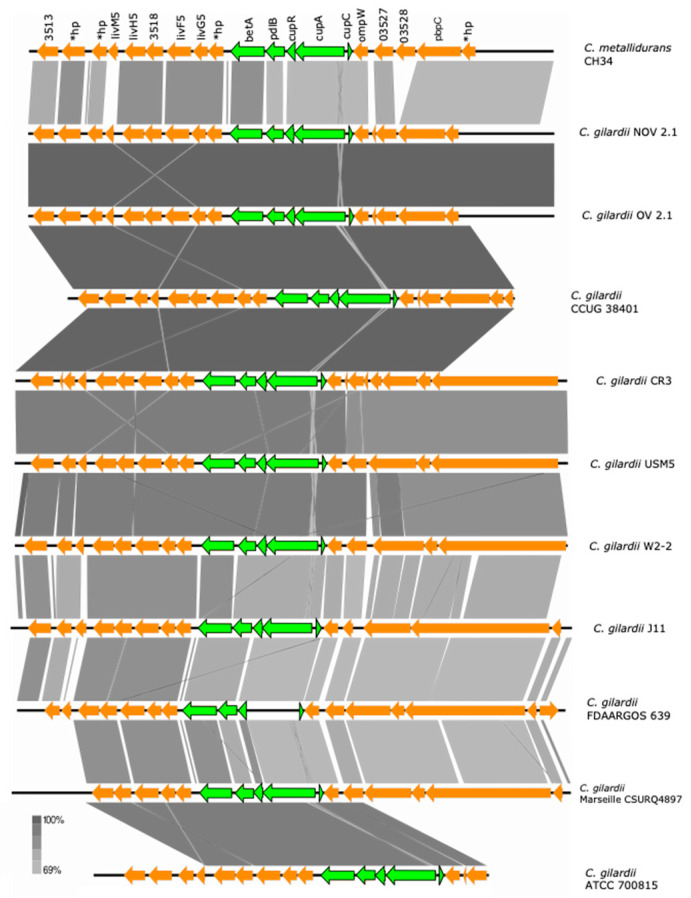
Map showing the genomic organization of the *cup* cluster in *C. metallidurans* CH34, NOV2-1, OV2-1, and other *C. gilardii* strains. The conserved and non-conserved genes of the cluster are colored in neon and olive green, respectively. Orange arrows indicate neighboring genes. The genes colored in brown indicate parts of horizontal DNA transfer-related sequences. *hp means hypothetical protein. The gray scale indicates the levels of synteny.

This cluster and the *betA2*/*pldB* adjacent genes were found to be highly conserved in NOV2-1, OV2-1, and other *C. gilardii* strains except in FDAARGOS639 where the *cupA* gene was missing (Figure 6). The *cup* proteins of NOV2-1 and OV2-1 had amino acid identities of 66.4–83.5% to those of *C. metallidurans* CH34 (Table S5). No horizontal DNA transfer sequences were detected at the proximity of the *cup* cluster in *C. metallidurans* CH34 and the *C. gilardii* strains analyzed here.

#### 3.5.6. *mmf* Cluster

*C. metallidurans* CH34 has three identical copies of the *mmf* cluster (“multi metal phenotype”), one in chromosome 1 and two in chromosome 2 (chromid). The *mmf* cluster consists of eight genes contained in the Tn6048 transposon (*mmfA*: propeptide, PepSY, and peptidase M4 precursor; *mmfB*: undecaprenyl pyrophosphate phosphatase; *mmfC1*: putative MFS permease; *mmfD*: secreted protein; *mmfC2*: putative MFS permease; *mmfR*: two-component transcriptional regulator; *mmfS*: periplasmic sensor signal transduction histidine kinase; *eamA*: EamA family transporter). This cluster is upregulated in the presence of Pb and Zn [36].

**Figure 7:**
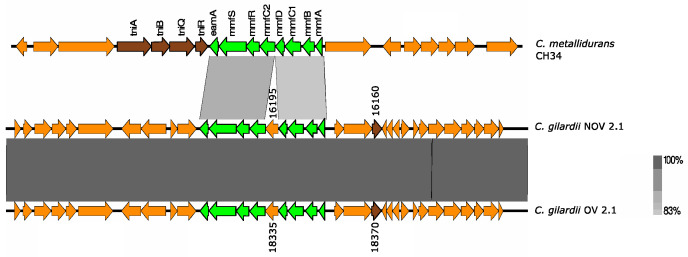
Map showing the genomic organization of the *mmf* cluster in *C. metallidurans* CH34, NOV2-1, OV2-1, and other *C. gilardii* strains. The conserved and non-conserved genes of the cluster are colored in neon and olive green, respectively. Orange arrows indicate neighboring genes. The genes colored in brown indicate parts of horizontal DNA transfer-related sequences. The gray scale indicates the levels of synteny.

This cluster was only found in NOV2-1 and OV2-1 but not in the other *C. gilardii* genomes used in this study (Figure 7). The *mmf* gene products had a higher amino acid sequence identity (74.5–95.6%) than those found on the *C. metallidurans* CH34 genome (Table S5). The horizontal DNA transfer sequences in the vicinity of the *mmf* cluster were different in *C. gilardii* and in *C. metallidurans* CH34 (Figure S7).

#### 3.5.7. *mer* Cluster

*C. metallidurans* CH34 has three copies of the *mer* cluster, one copy in each plasmid (in the Tn4378 transposon of pMOL28 and the Tn4380 transposon of pMOL30) and one copy in chromosome 1. The *mer* cluster on chromosome 1 consists of 3 genes (*merT*: mercury ion transport protein; *merP*: periplasmic mercury ion-binding protein; *merA*: mercury reductase). The *merA* gene encodes a reductase that participates in the demethylation of organic mercury compounds [40].

On pMOL28 and pMOL30, the cluster contains two additional genes, *merD* and *merE*, which encode, respectively, a regulatory protein involved in Hg resistance and a broad spectrum organic and inorganic Hg transporter [36,40]. In all cases, a *merR* gene encoding a regulatory protein is found upstream *merT*; however, this gene may not be part of the *mer* cluster [36]. The *mer* genes (except *merR*) are fully induced by Hg and partially activated by Cd, Zn, and Pb [36].

**Figure 8:**
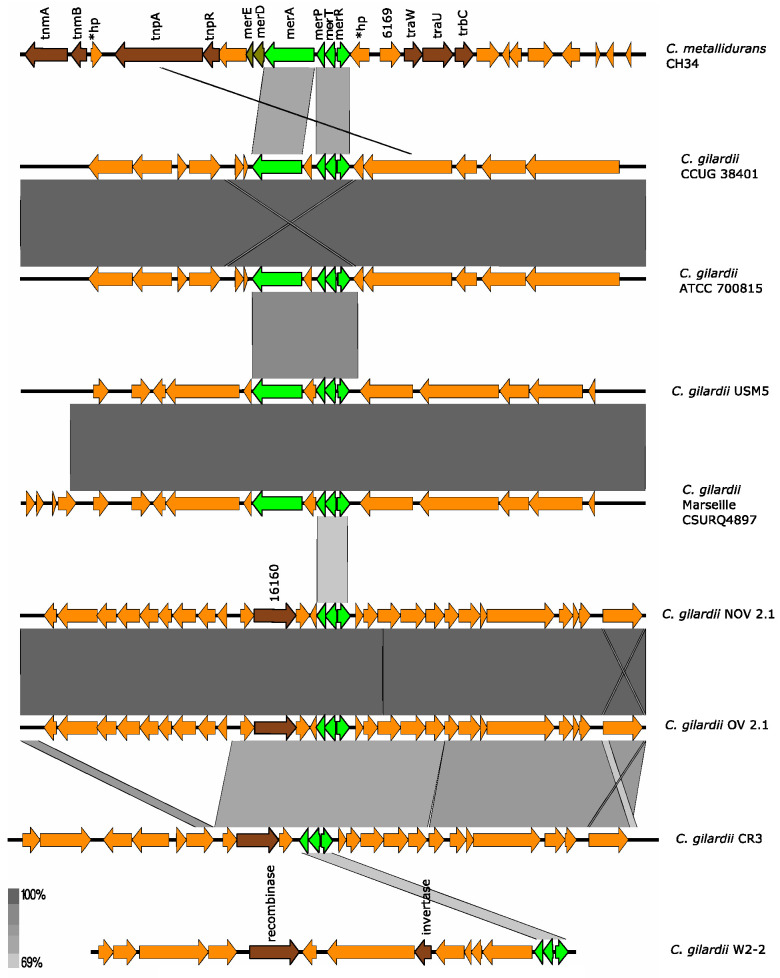
Map showing the genomic organization of the *mer* cluster in *C. metallidurans* CH34, NOV2-1, OV2-1, and other *C. gilardii* strains. The conserved and non-conserved genes of the cluster are colored in neon and olive green, respectively. Orange arrows indicate neighboring genes. The genes colored in brown indicate parts of horizontal DNA transfer-related sequences. *hp means hypothetical protein. The gray scale indicates the levels of synteny.

None of the *C. gilardii* genomes used here harbored the complete *mer* cluster. In *C. gilardii* CCUG38401, ATCC 700815, USM5, and Marseille CSURQ4897 only the *merP*, *merT*, *merR*, and *merA* genes were found while only *merP*, *merT*, and *merR* were found in NOV2-1, OV2-1, *C. gilardii* CR3, and W2-2 (Figure 8). The *mer* cluster was completely absent in *C. gilardii* FDAARGOS 639 and J11. The amino acid identity of the *mer* gene products of NOV2-1 and OV2-1 were 62-71% (Table S5).

## 4. Discussion

*C. gilardii* was first retrieved from environmental and clinical samples [41]. Initially named *Wautersia gilardii* and then *Ralstonia gilardii*, *W. gilardii*, together with all the other members of the genus *Wautersia* were later reclassified into *Cupriavidus* [12]. Since then, *C. gilardii* has been isolated from water, agricultural soil contaminated with the 2,4-dichlorophenoxyacetic acid herbicide, soils contaminated with heavy metals and asphalts, as well as human clinical samples [18,42,43]. *C. gilardii* has potential biotechnological applications in the detoxification of heavy metals and biodegradation of recalcitrant compounds, such as naphthenic acids and 2,4-dichlorophenoxyacetic acid [18,44,45,46]. It is also considered an emerging multidrug-resistant pathogen found in many environments [47].

Here, we sequenced the genomes of two *C. gilardii* strains, NOV2-1 and OV2-1, isolated from an iron oxide crust sampled in a tunnel of the Naica mine [8,11]. Unique characteristics of this biotope are the thermophilic environment (45–55 °C) and the presence of heavy metals [1]. NOV2-1 and OV2-1 had initially been identified as *C. taiwanensis* by partial 16S rRNA gene sequencing [8,11]. The standard ANI cut-off value for considering genomes to be from the same species is 95–96% [48]. For *Cupriavidus*, ANI cut-off values of 90% have been proposed [49]. As NOV2-1 and OV2-1 showed ANI values > 98% with *C. gilardii* CCUG 38401^T^ (Figure 1a and Table S2), it can be concluded that these strains belong to the *C. gilardii* species. The phylogenetic tree based on 510 concatenated core genes also clearly showed that NOV2-1 and OV2-1 grouped with *C. gilardii* species rather than with *C. taiwanensis* (Figure 1b). The phylogenetic tree and the ANI values for *Cupriavidus cauae* USM5 (90.62% of ANI with CCUG 38401) and *Cupriavidus* sp. HPC(L) (94.4% of ANI with CCUG 38401) indicated that these strains should be reclassified as *C. gilardii*. The reclassification of *Cupriavidus* sp. HPC(L) into the *C. gilardii* species had already been proposed from a previous comparative genomic analysis [47]. *C. gilardii* J11, which was located on a separate branch in the phylogenetic tree and showed an ANI value of 82.84% with CCUG 3840^T^, may be a new *Cupriavidus* species. These results again highlight the complexity of the *Cupriavidus* taxonomy [12,13].

The genomes of NOV2-1 and OV2-1 consisted of two chromosomes (chr1: 3.58 Mb and chr2: 2.1 Mb for NOV2-1 and chr1: 3.53 Mb and chr2: 2.1 Mb for OV2-1) summing, respectively, 5.67 and 5.64 Mb (Table 1). These values as well as the GC and CDS content fall in the range reported for other *C. gilardii* complete genomes (Table S1). No plasmids were found in NOV2-1 and OV2-1, although small replicons of 33,330 and 87,261 bp have been reported in other *C. gilardii* strains, such as FDAARGOS_639 and QJ1. The chromosome 1 of both strains displayed *dnaA-dnaN-gyrB* gene structures (locus tags: K6V71_17740-50 in NOV2-1 and K7A44_16775-85 in OV2-1) while chromosome 2 presented a conserved *repA-parA-parB* region indicating a plasmid-like replication and partitioning mechanisms (locus tags: K6V71_04650-65 in NOV2-1 and K7A44_04730-45 in OV2-1). Additionally, 24 of the 510 core genes were located on chromosome 2 in both strains (Table S6) of which 13 were related to motility (chemotaxis proteins and flagellum synthesis) and the other to amino acids catabolism, gluconate utilization (permease and Entner-Doudoroff enzyme), cell wall biosynthesis and breakdown of aromatic compounds. The combination of a few core genes and plasmid-like replication origin indicates that chromosome 2 is a chromid [50,51]. This is not surprising since *Cupriavidus* and *Ralstonia* are known to harbor a secondary chromosome designated a chromid, i.e., a replicon that is neither a chromosome nor a plasmid [49,52,53,54]. These results are consistent with those obtained by [55], which analyzed the chromids present in nine *Cupriavidus* strains from several species and proposed that these secondary replicons allow the cells to expand their genome content through horizontal gene transfer and adapt to novel environments.

As the Naica mine is a thermophilic biotope, the influence of temperature on the growth of NOV2-1 and OV2-1 was evaluated. These strains had their maximum specific growth rate (μmax) at 42 °C and showed growth capacity up to 48 °C as *C. gilardii* CCUG 38401^T^ (Figures S1 and S2). *C. metallidura*ns CH34, *C. necator* LMG 8453, and *C. taiwanensis* LMG19424 could not grow at temperatures above 37 °C. These results indicate that the capacity to grow at higher temperatures (>40 °C) could be a phenotypic characteristic of the *C. gilardii* species, although more studies with other *C. gilardii* strains are needed to confirm this assumption. Concerning temperature, *C. metallidurans* CH34 displays an intriguing temperature-induced mutagenesis and mortality phenotype when temperature is shifted from 30 °C to 37 °C [56]. This phenotype has been recently attributed to a “genetic defect” at the *lysA* locus, which encodes the diaminopimelate decarboxylase gene, part of the peptidoglycan biosynthetic pathway [57]. Using the *C. metallidurans* CH34 Rmet_6588- *lysA* sequence as the query in a Blastn search against *C. gilardii* CR3, OV2-1, and NOV2-1, we found that the CH34 sequence presented important differences with its *C. gilardii* counterparts, specifically gaps and inversions in the 120 bp upstream the ATG codon of the Rmet_6588 gene. This gene putatively encodes a lipoprotein. These differences might influence the expression of the lipoprotein and the synthesis of peptidoglycan and explain the growth of *C. gilardii* strains above 37 °C. Detailed genomic studies and experiments are needed to study this phenotype.

The *Cupriavidus* strains used in this study were unable to assimilate five or six carbon carbohydrates and sugar alcohols (Table S3). These results are consistent with the description of other members of the *Cupriavidus* genus [12,16,23,58]. Such a carbohydrate utilization pattern has been found advantageous for the microbial detoxification of lignocellulosic biomass hydrolysates where the detoxifying microorganism selectively metabolizes lignocellulose-derived microbial inhibitors without compromising the fermentable sugars fraction [59]. This strategy has been reported with *Cupriavidus basilensis* for the removal of furans and phenolic compounds in acid-pretreated *Miscanthus giganteus* hydrolysates [60].

Regarding heavy metals tolerance, *C. metallidurans* CH34 was the most tolerant bacteria, which is consistent with this strain, the model organism to study heavy metal resistance and tolerance [61]. Several gene clusters involved in heavy metal homeostasis (*ars*, *czc*, *cop* 1, *sil*-*cop 2*, *cup*, *mmf*, and *mer*) were detected, fully or partially conserved, in NOV2-1, OV2-1, and other *C. gilardii* genomes (Figure 3, Figure 4, Figure 5, Figure 6, Figure 7 and Figure 8). The presence of HMR clusters in the NOV2-1 and OV2-1 genomes were related to the heavy metal tolerance assay, where NOV2-1 and OV2-1 presented a high tolerance to heavy metals (Ni, Co, Cu, Cd, Zn, and Pb ions) at the millimolar levels in a LB-Lennox buffered solid medium (Figure 2), indicating that these strains can orchestrate a protective process against high levels of these metal ions.

OV2-1 was more sensitive to Cd and Zn than NOV2-1 and *C. gilardii* CCUG 38401^T^. This could be because the *czc* cluster was not found on the genome of OV2-1 contrary to NOV 2-1 and *C. gilardii* CCUG 38401^T^, which possessed an almost complete *czc* cluster (*czcABCDEIJMNRS*) (Figure 4) with a 98-100% of amino acid identity with the *C. metallidurans* CH34 *czc* gene products (Table S5). This cluster consists of eleven genes, including *czcMNICBADRSEJP*, which have been reported to be important for Co, Zn, and Cd tolerance in *C. metallidurans* CH34 [37]. It encodes three different efflux systems: (1) a cation diffusion facilitator CzcD, (2) a heavy metal Resistance–Nodulation–Division (RND)-driven system CzcCBA, and (3) a cation efflux P1B4-type ATPase CzcP that transports ions from the cytoplasm to the periplasm [37,62,63]. This system shields the cytoplasm against high extracellular concentrations of Zn, Co, and Cd ions [64]. The deletion of the *czcA* and *czcB* genes results in the complete loss of ions efflux and sensitivity to Cd, Co, and Zn in *C. metallidurans* [37]. The *czc*P gene was absent in NOV2-1 and no differences in tolerance in Zn, Cd, and Co tolerance were observed between this strain and *C. gilardii* CCUG 38401^T^ (Figure 2), which supports the role of CzcP as a metal resistance enhancer [37].

Other ATPases from the P1B2 type, such as CadA and ZntA, are also involved in Zn and Co resistance in *C. metallidurans* CH34 [65]. Using the *C. metallidurans* CH34 ZntA sequence as the query in a Blastp search, possible homologs of this protein were found in the NOV2-1 genome (locus tag K6V71_07115, coverage: 91%, E-value: 0, identity: 74.8%) and in the OV2-1 genome (locus tag K7A44_07215, coverage: 89%, E-value: 0, identity: 75%). No homologues of CadA were found in NOV2-1 and OV2-1. The absence of homologs of CadA, CzcP, and the Pbr system in NOV2-1 and OV2-1 could be the limiting factor for these strains to tolerate higher concentrations of Cd, Zn, and Pb. However, detailed transcriptomic and proteomic analyses are needed to confirm this hypothesis.

In *C. metallidurans*, the *czc* cluster is flanked by tyrosine-type recombinase/integrase (upstream) and IS3 transposase family (downstream) sequences related to the mobility of this cluster [66]. These sequences were partially conserved in NOV2-1, however two additional and identical IS3-related insertion sequences were also detected, one inside the cluster between the *czcI* and *czcN* genes (locus tag K6V71_25340) and one on the right side of the cluster (locus tag K6V71_25285) (Figure S4). A nucleotide blast analysis showed that this insertion sequence was 100% identical to a sequence in the genome of a *Cupriavidus campinensis* strain (MJ1). The high degree of sequence identity of the *czc* proteins and the presence of conserved sequences related to horizontal DNA transfer suggests a horizontal transmission of this cluster among the *Cupriavidus* genus.

The *cop* 1, *cop* 2, and *cup* clusters were found in NOV2-1, OV2-1, and other *C. gilardii* genomes (Figure 5 and Figure 6). These clusters are the three major Cu detoxification systems in *C. metallidurans* CH34. Among them, the *cop* cluster 1 only responds to Cu ions, while the *cop* cluster 2 responds to Cd, Cu, Ni, Zn, Pb, Ag, and Co. Even though NOV2-1 had homologs of these clusters in its genome, this strain was slightly more sensitive to Cu than OV2-1 and CCUG38401^T^ indicating that some genes or proteins in these clusters may not be functional or that other genes are needed for optimal Cu resistance. Surprisingly, a homologous *mmf* cluster was found both in *C. gilardii* NOV2-1 and OV2-1 but not in the other *C. gilardii* strains used in this study (Figure 7). Nevertheless, no growth differences were observed in the presence of Cd, Co, and Zn between NOV2-1 and CCUG38401^T^; these results could indicate that the function of this cluster can be substituted by other HMR proteins and systems. Although this cluster is strongly induced in the presence of Zn and Pb, its function is still unknown [67]. Furthermore, an incomplete *mer* cluster was found in NOV2-1, OV2-1, and other *C. gilardii* genomes, being the *merP* (periplasmic binding protein), *merT* (mercuric ion transport) and *merR* (transcriptional regulator) conserved genes (Figure 8). However, the functionality of these incomplete clusters during heavy metal exposure in *C. gilardii* NOV2-1, and OV2-1, and other *C. gilardii* strains has to be verified. Different or no horizontal DNA transfer sequences were present around these HMR gene clusters in *C. gilardii* compared to *C. metallidurans*, their role in the mobilization and expression of HMR genes in the *Cupriavidus* genus requires additional studies (Figures S5–S8).

Finally, an incomplete *ars* cluster homologue, lacking the *arsC*2 (arsenate reductase), *arsI* (lactoylglutathione lyase), *arsM* (methyltransferase), and *arsP* (permease) genes, was found in NOV2 -1, OV2-1, and other *C. gilardii* genomes (Figure 3). From a physiological point of view, the main genes for arsenic detoxification are present: *arsR* (transcriptional regulator), *arsB* (efflux transporter), *arsC*1 (reductase), and *arsH* (resistance protein). Although As was not studied here, the presence of this metal has been reported in Naica’s water and in the Meoqui-Delicias aquifer [4,5]. The presence of this cluster could reflect the presence of As; however, it has been reported that *ars* genes are also present in microorganisms isolated in As-free habitats [68]. A recent survey of arsenic-related genes in soil microbiomes has revealed a potential vertical and horizontal transfer history of the *ars* genes [69].

## 5. Major Implications and Future Perspectives

The findings from this study have important implications for understanding the genetic and physiological characteristics of the *C. gilardii* species, especially in the context of environmental adaptability and bioremediation potential. The genomic analysis of the strains NOV2-1 and OV2-1, isolated from the thermophilic and heavy metal containing environment of the Naica mine, revealed the capacity of this species to withstand both high temperature and the presence of metals. It has been shown that metals availability and, therefore, toxicity increases as temperature rises [70]. The high tolerance of these strains to multiple heavy metals, such as nickel, cobalt, copper, cadmium, zinc, and lead, suggests that *C. gilardii* could be an important player in the bioremediation of contaminated sites at elevated temperatures, especially in the context of climate change. Research, both at the genomic and experimental level, on the origin, evolution, mobility, and expression of HMR gene clusters in the *Cupriavidus* genus should continue, taking advantage of the increasing number of strains and completely sequenced genomes from different environments. This work also raises new research opportunities on temperature adaptation mechanisms with the *Cupriavidus* taxon and its relationship with HMR.

## 6. Conclusions

This study clarifies the taxonomic classification of two novel *Cupriavidus* strains, NOV2-1 and OV2-1, isolated from a unique, thermophilic, heavy metal-rich environment within the Naica mine located in Chihuahua, Mexico. Through comparative genomic analyses, we conclusively reclassified these strains as *C. gilardii*. Moreover, the reclassification of other *Cupriavidus* strains is also proposed here, underscoring the complexity of the *Cupriavidus* taxonomy. Furthermore, our genomic analysis revealed the presence of a secondary chromosome, or chromid, in both NOV2-1 and OV2-1, a characteristic feature of the *Cupriavidus* genus. Physiologically, these strains exhibit thermotolerance at temperatures up to 48 °C, a trait potentially characteristic of *C. gilardii*. Nutritionally, they share a similar carbohydrate utilization pattern with other *Cupriavidus* species, potentially advantageous in lignocellulosic biomass detoxification. Finally, our study provides insights into the genetic basis of heavy metal tolerance in these strains. The presence and differential conservation of HMR gene clusters, including *czc*, *cop*, *cup*, *mmf*, *mer*, and *ars*, correlate with the observed tolerance levels to various heavy metals. Specifically, the absence of a complete *czc* cluster in OV2-1 likely contributes to its increased sensitivity to Cd and Zn. The presence of mobile genetic elements surrounding these HMR clusters suggests horizontal gene transfer as a key mechanism for the dissemination and expression of HMR capacity within *Cupriavidus* populations. This study also highlights the potential of these strains for biotechnological applications in bioremediation, heavy metal detoxification, and biomass hydrolysate detoxification.

## Figures and Tables

**Table 1:** General genomic features of NOV2-1 and OV2-1.

Feature	NOV2-1	OV2-1
Genome size (bp)	5,687,442	5,647,760
CDS	5024	5011
rRNA	12	12
tRNA	59	59
GC content (%)	67.53	67.58
Contigs number	2	2
Chromosomes number	2	2
Hypothetical proteins	1186	1186
Proteins with functional assignments	4095	4064

**Table 2:** Summary of physiological characteristics of strains NOV2-1 and OV2-1 compared to the *Cupriavidus* reference strains used in this work. The complete results of the 47 biochemical tests included in the Vitek 2 Gram-negative identification card are shown in Table S4.

Characteristics	*C. necator*	*C. metalidurans*	*C. taiwanensis*	*C. gilardii*		
	LMG8453	CH34	LMG19424	CCUG 38401	NOV2-1	OV2-1
Range for growth:						
Temperature (°C)	30–37	30–35	30–37	30–48		
pH	5.0–9.0			7.0–9.0	7.0–9.9	5.5–9.0
NaCl (% *w*/*v*)	>1			<1		
Heavy metal tolerance (mM):						
Cd	1.5	5	0.75	1.5	5	0.75
Co	0.5	0.8	0.8	0.7	0.7	0.7
Cu	3	3	1.5	3	1	3
Pb	8.5	9	8.5	9	8.5	8.5
Ni	3	7	3	7	3	3
Zn	2	7.5	7.5	7.5	7.5	1.5
Assimilation of:						
L-Lactate	+	−	+	−	+	+
Sodium citrate	+	+	+	−	−	+
L-Malate	-	+	-	-	+	+
Enzymatic activities:						
L-Proline Arylamidase	+	+	+	−	−	−
L-Pyrrolydonyl-Arylamidase	+	+	+	−	−	−
Gamma-Glutamyl-Transferase	+	−	+	−	−	−
Urease	−	+	+	−	−	−
Alpha-Glucosidase	−	−	−	−	−	−
Glutamyl Arylamidase pNA	+	−	−	+	−	+
Other tests:						
ELLMAN	+	+	+	+	−	−

+ means positive for the test and − means negative for the test.

## Data Availability

The chromosomes and chromids sequences of *C. gilardii* NOV2-1 and OV2-1 can be accessed under the GenBank accession number CP083437.1, CP083438.1, CP083735.1 and CP083736.1 (https://www.ncbi.nlm.nih.gov/datasets/genome/GCF_025643215.1/ and https://www.ncbi.nlm.nih.gov/datasets/taxonomy/82541/, accessed on 22 January 2025). The sequencing data of PacBio and Illumina reads are available in the NCBI SRA database via the BioProject accession number PRJNA757420 (https://www.ncbi.nlm.nih.gov/bioproject/PRJNA757420/, accessed on 22 January 2025).

## Supplementary Materials

The following supporting information can be downloaded at: https://www.mdpi.com/article/10.3390/microorganisms13040809/s1, Figure S1: Growth of *Cupriavidus* Reference Strains and the NOV2-1 and OV2-1 Strains at Different Temperatures; Figure S2: Maximum Specific Growth Rate (μmax) of strains NOV2-1 and OV2-1 at different temperatures; Figure S3: Sequences Related to Horizontal DNA Transfer at the Viscinity of the *ars* Cluster in *C. metallidurans* CH34, NOV2-1 and OV2-1; Figure S4: Sequences Related to Horizontal DNA Transfer at the Viscinity of of the *czc* Cluster in CH34, NOV2-1 and OV2-1; Figure S5: Sequences Related to Horizontal DNA Transfer at the Viscinity of the *cop* 1 Cluster; Figure S6: Sequences Related to Horizontal DNA Transfer at the Viscinity of the *cop* 2 Cluster; Figure S7: Sequences Related to Horizontal DNA Transfer at the Viscinity of the *mmf* Cluster; Figure S8: Sequences Related to Horizontal DNA Transfer at the Viscinity of the *mer* Cluster; Table S1: Genome Structure Comparison Between NOV2-1, OV2-1, and other *C. gilardii* strains; Table S2: Strains and Isolates Used in Comparative Genomic Analyses; Table S3: Average Percentage Nucleotide Identity (ANI) Between Pairs of *Cupriavidus* genomes; Table S4: Results of Carbon Source Utilization, Inhibition and Resistance, and Enzymatic Activities Tests of *C. gilardii* NOV2-1, *C. gilardii* OV2-1 and *Cupriavidus* Reference Strains; Table S5: HMR Gene Clusters Detected in *C. gilardii* NOV2.1 and OV2.1; Table S6: Core Genes Found in the NOV2.1 and OV2.1 Chromids.
