# Supplementary material for: Physiology, Heavy Metal Resistance, and Genome Analysis of Two Cupriavidus gilardii Strains Isolated from the Naica Mine (Mexico)
Source: Microorganisms. 2025 Apr 2;13(4):809. doi: 10.3390/microorganisms13040809 (PMC12029693; doi:10.3390/microorganisms13040809)
Supplement: Supplementary file 1 [file microorganisms-13-00809-s001.zip › microorganisms-3527095-supplementary Figure S1-S8.pdf]

Figure S1. Growth of *Cupriavidus* reference strains and the NOV2-1 and OV2-1 strains at different temperatures.

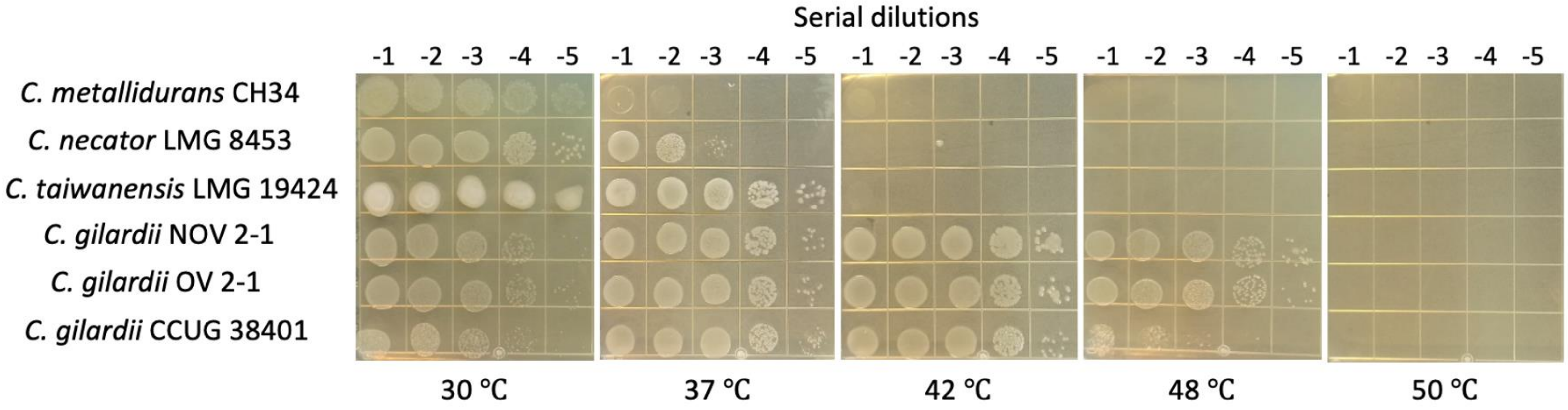

Figure S2. Maximum specific growth rate (µmax) of strains NOV2-1 and OV2-1 at different temperatures.

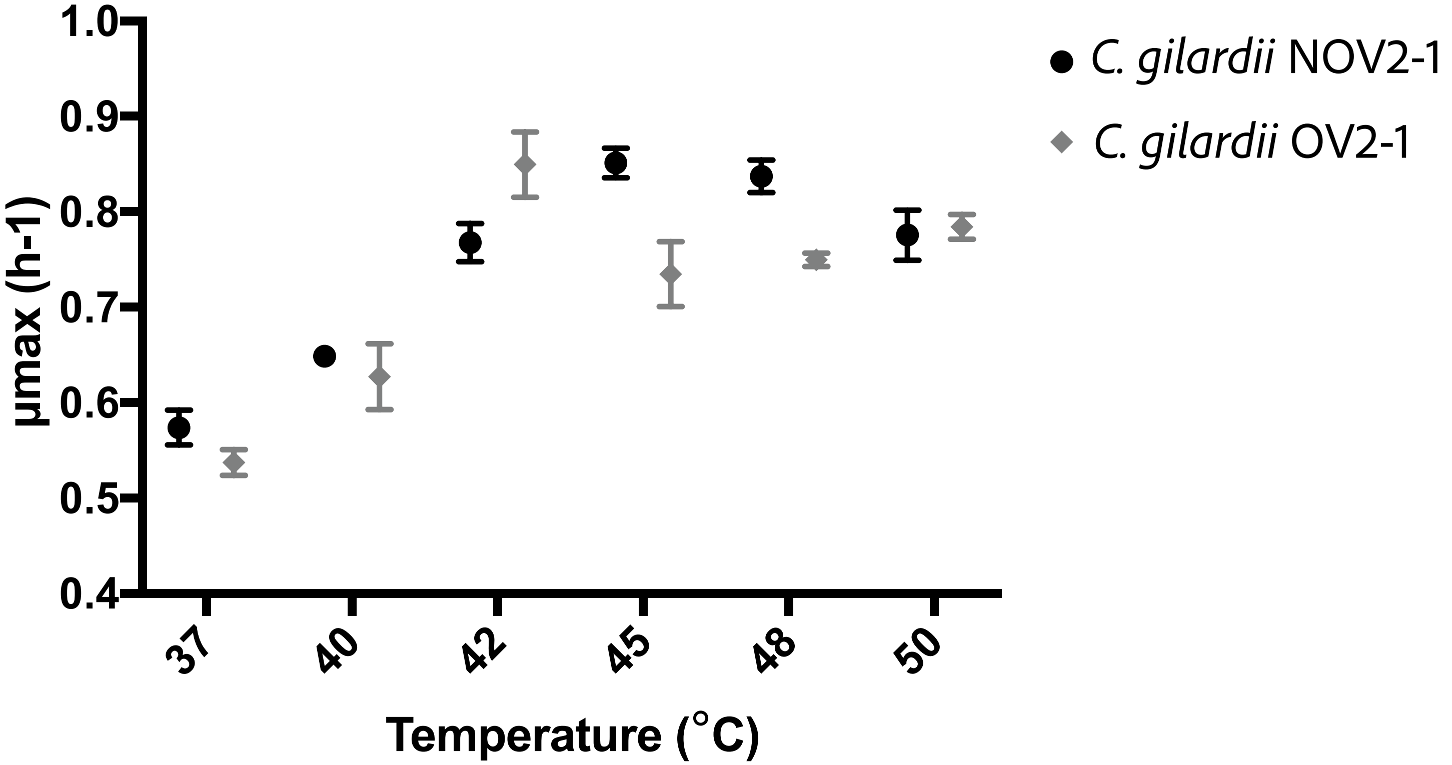

Figure S3. Sequences related to horizontal DNA transfer at the viscinity of the *ars* cluster in *C. metallidurans* CH34, NOV2-1 and OV2-1.

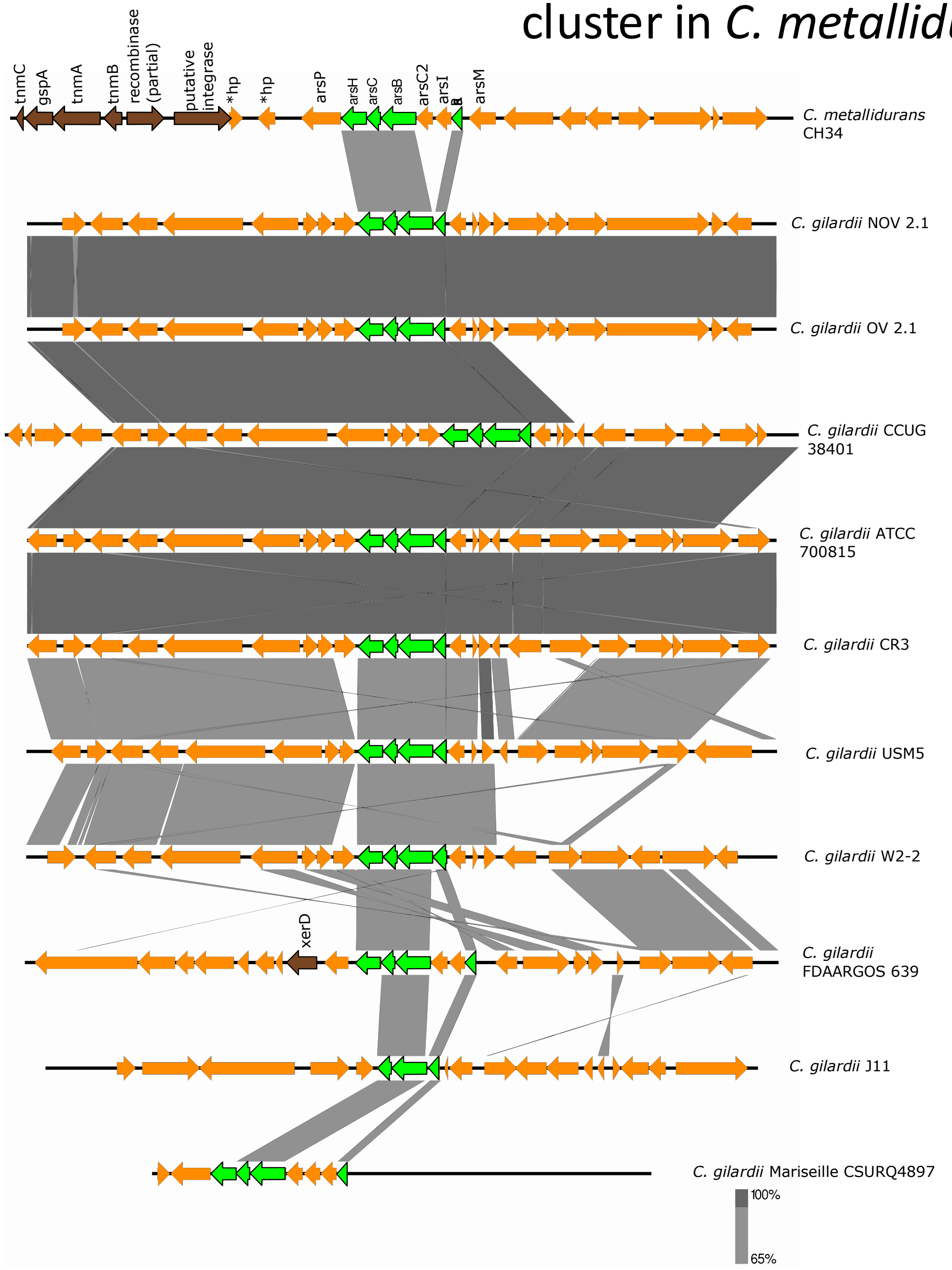

	CH34 Locus tag/Gene	Gene Anotation	Locus tag in NOV 2.1	Locus tag in OV 2.1	NCBI gene anotation
Horizontal DNA transfer related	Rmet_0319/tnmC	conserved hypothetical protein Tn6049			
	Rmet_0320/gspA	ATPase (type II general secretion pathway) Tn6049			
	Rmet_0321/tnmA	transposase Tn6049			
	Rmet_0322/tnmB	protein involved in mobility of Tn6049			
	Rmet_0323	tyrosine-based site specific recombinase (partial)			
	Rmet_0324	putative integrase			
ars system/As-Pb-Zn-Se-Co-Cd	Rmet_0327/arsP	permease of the Major Facilitator Family			
	Rmet_0328/arsH	NADPH-dependent FMN reductase	K6V71_09305	K7A44_25200	arsenical resistance protein ArsH
	Rmet_0329/arsC	arsenate reductase	K6V71_09300	K7A44_25205	arsenate reductase (glutaredoxin)
	Rmet_0330/arsB	bile acid:sodium symporter	K6V71_09295	K7A44_25210	ACR3 family arsenite efflux transporter
	Rmet_0331/arsC2	arsenate reductase (glutaredoxin family) CMGI-7			
	Rmet_0332/arsI	Lactoylglutathione lyase (glyoxalase family) CMGI-7			
	Rmet_0333/arsR	ArsR family transcriptional regulator	K6V71_09290	K7A44_25215	metalloregulator ArsR/SmtB family transcription factor
	Rmet_0334/arsM	S-adenosyl-L-methionine-dependent methyltransferase			

Figure S4. Sequences related to horizontal DNA transfer at the viscinity of of the *czc* cluster in CH34, NOV2-1 and OV2-1.

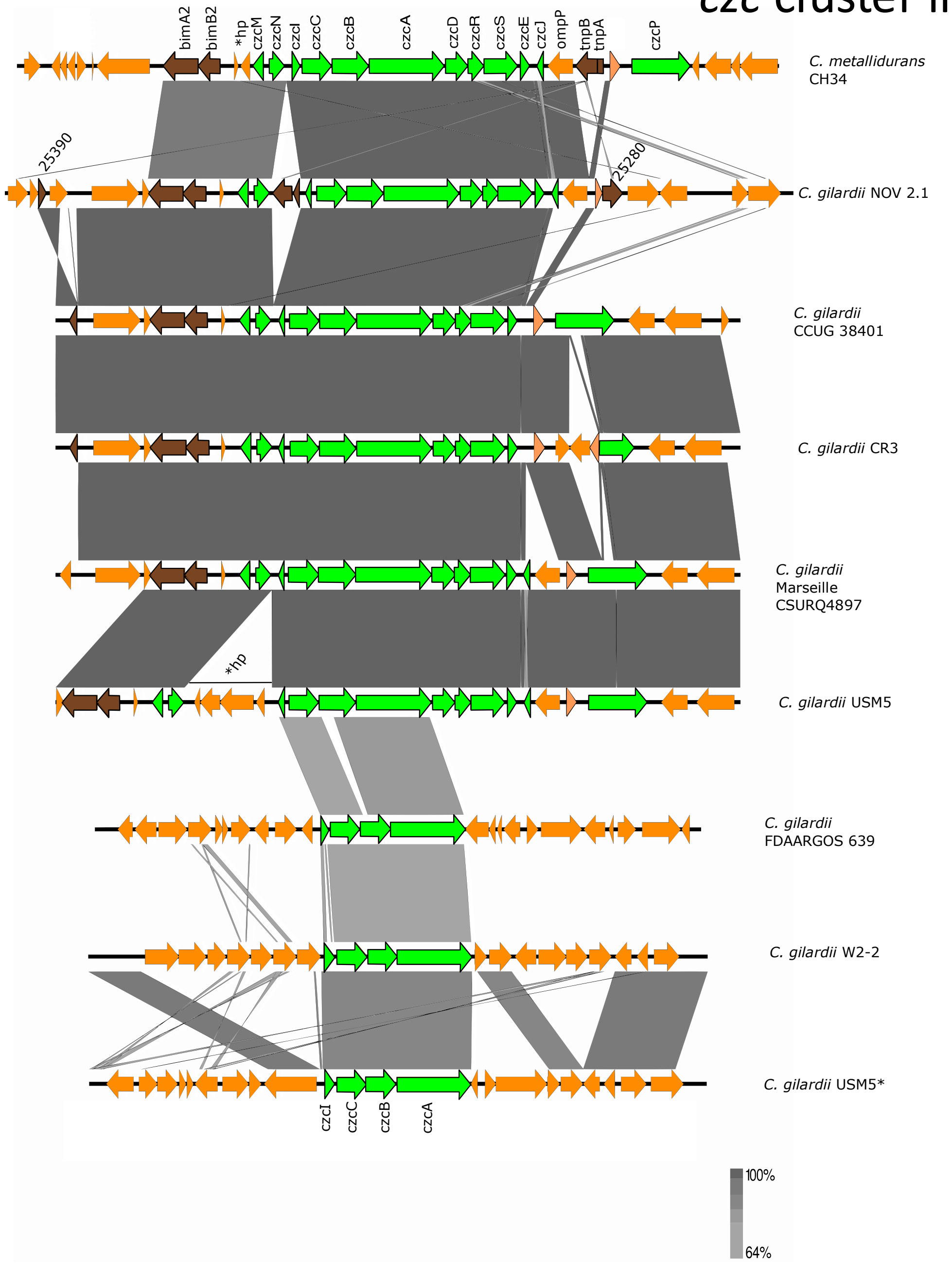

	CH34 Locus tag/Gene	Gene Anotation	Locus tag in NOV 2.1	NCBI gene anotation
Horizontal DNA transfer related			K6V71_25390	IS3-like element ISRme15 family transposase
	Rmet_5989/bimA2	tyrosine-type recombinase/integrase	K6V71_25370	tyrosine-type recombinase/integrase
	Rmet_5988/bimB2	DNA-binding protein	K6V71_25365	DNA-binding protein
	Rmet_5987	hypothetical protein	K6V71_25360	hypothetical protein
	Rmet_5986	hypothetical protein	K6V71_25355	hypothetical protein
czc system/Co-Zn-Cd	Rmet_5985/czcM	MgtC/SapB family	K6V71_25350	MgtC/SapB family protein
	Rmet_5984/czcN	heavy metal efflux RND transporter	K6V71_25345	heavy metal efflux RND transporter CzcN
			K6V71_25340	IS3 family transposase
	Rmet_5983/czcI	CDF family cobalt-zinc-cadmium transporter CzcI	K6V71_25335	CDF family cobalt-zinc-cadmium transporter CzcI
	Rmet_5982/czcC	outer membrane protein	K6V71_25330	heavy metal efflux RND transporter CzcC
	Rmet_5981/czcB	membrane fusion protein	K6V71_25325	heavy metal efflux RND transporter CzcB
	Rmet_5980/czcA	integral membrane protein	K6V71_25320	heavy metal efflux RND transporter CzcA
	Rmet_5979/czcD	Cation efflux protein (CDF)	K6V71_25315	cation diffusion facilitator family transporter
	Rmet_5978/czcR	Regulator two-component regulatory system	K6V71_25310	heavy metal homeostasis two-component system response regulator CzcR
	Rmet_5977/czcS	Sensor two-component regulatory system	K6V71_25305	sensor histidine kinase CzcS
	Rmet_5976/czcE	copper-binding protein	K6V71_25300	copper-binding periplasmic protein CzcE
	Rmet_5975/czcJ	Hypotetical protein	K6V71_25295	hypothetical protein
	Rmet_5974/ompP	porin	K6V71_25290	porin
Horizontal DNA transfer related	Rmet_5973/tnpB-A	IS3-like element ISRme13 family transposase		
			K6V71_25285	IS3 family transposase
			K6V71_25280	tyrosine-type recombinase/integrase

Figure S5. Sequences related to horizontal DNA transfer at the viscinity of the *cop* 1 cluster.

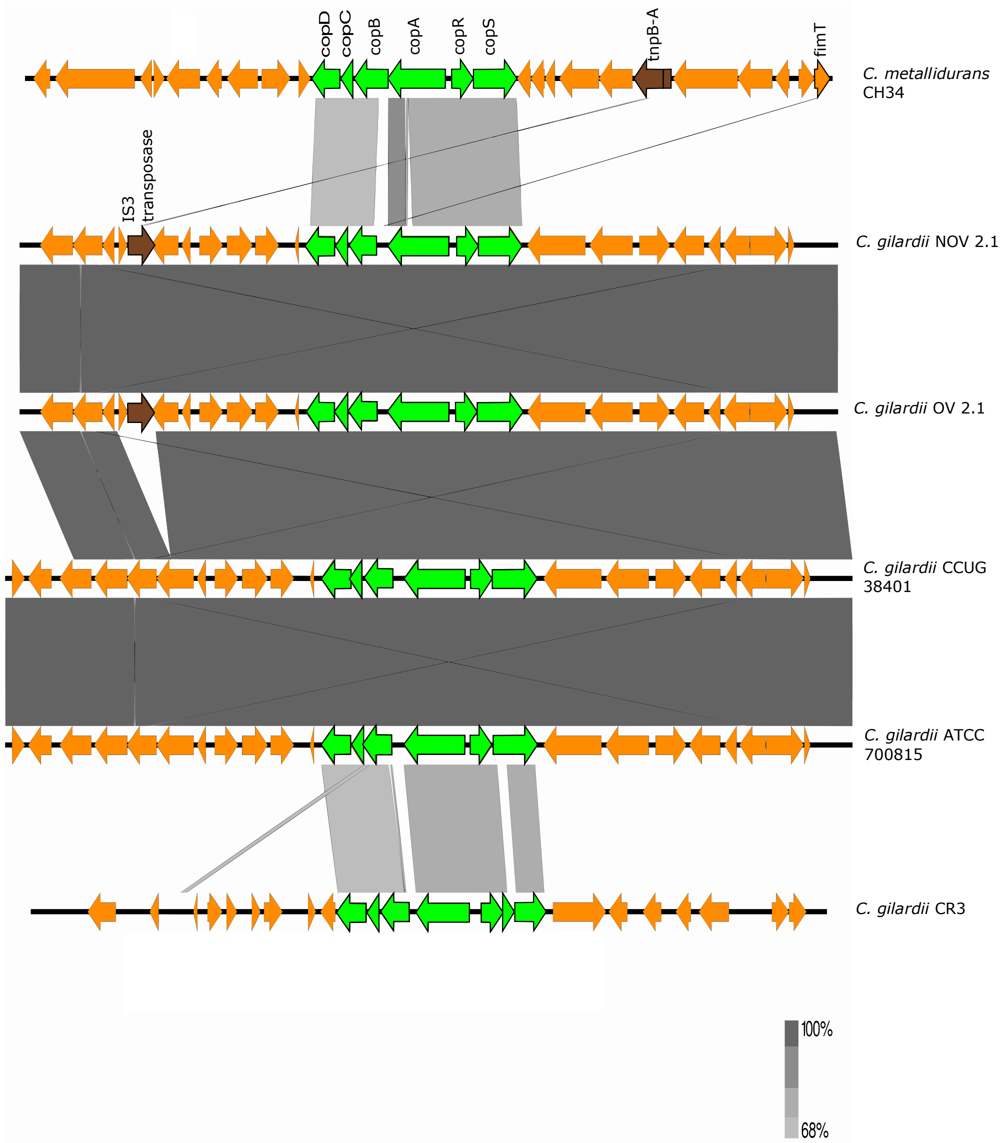

	CH34 Locus tag/Gene	Gene Anotation	Locus tag in NOV 2.1	Locus tag in OV 2.1	NCBI gene anotation
cop system /Cu	Rmet_5668/copD	copper homeostasis membrane protein CopD	K6V71_18740	K7A44_15770	copper homeostasis membrane protein CopD
	Rmet_5669/copC1	copper resistance protein CopC	K6V71_18745	K7A44_15775	copper homeostasis periplasmic binding protein CopC
	Rmet_5670/copB1	copper resistance B precursor	K6V71_18740	K7A44_15780	copper resistance protein B
	Rmet_5671/copA1	copper-resistance protein CopA	K6V71_18735	K7A44_15785	copper resistance system multicopper oxidase
	Rmet_5672/copR1	two component heavy metal response transcriptional regulator	K6V71_18730	K7A44_15790	heavy metal response regulator transcription factor
	Rmet_5673/copS1	heavy metal sensor signal traduction histidine kinase	K6V71_18725	K7A44_15795	heavy metal sensor histidine kinase
Horizontal DNA transfer related	Rmet_5679/tnpB-A	IS3-like element ISRme13 family transposase			
			K6V71_18785	K7A44_15735	IS3 family transposase

Figure S6. Sequences related to horizontal DNA transfer at the viscinity of the *cop* 2 cluster.

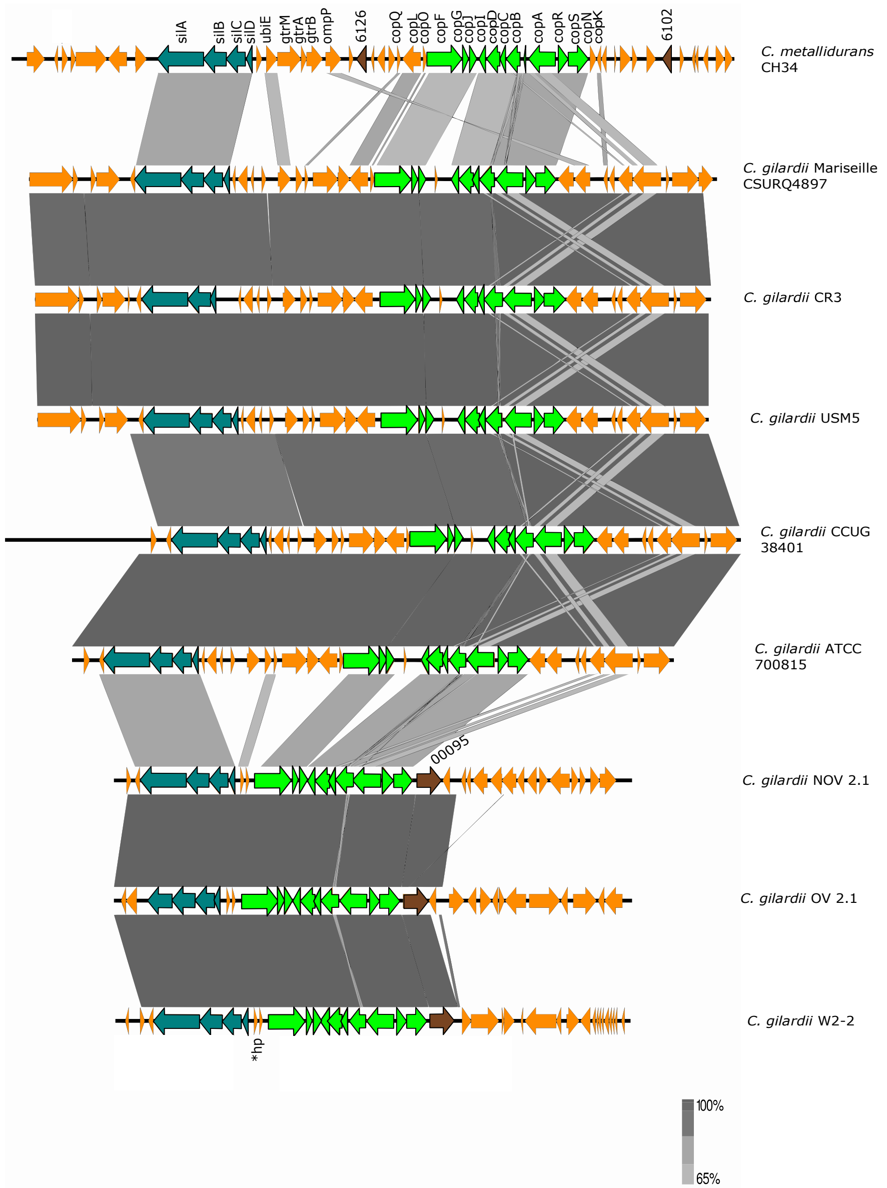

	CH34 Locus tag/Gene	Gene Anotation	Locus tag in NOV 2.1	Locus tag in OV 2.1	NCBI gene anotation
sil system/Ag	Rmet_6136/silA	proton antiporter metal efflux system	K6V71_00015	K7A44_00015	efflux RND transporter permease subunit
	Rmet_6135/silB	proton antiporter metal efflux system	K6V71_00020	K7A44_00020	efflux RND transporter periplasmic adaptor subunit
	Rmet_6134/silC	Outer membrane silver efflux protein	K6V71_00025	K7A44_00025	TolC family protein
	Rmet_6133/silD	copper/silver resistance protein SilD	K6V71_00030	K7A44_00030	copper resistance protein
	Rmet_6131/ubiE	class I SAM-dependent methyltransferase			
			K6V71_00035	K7A44_00035	CzcE family metal-binding protein
			K6V71_00040	K7A44_00040	hypothetical protein
cop system/Cu-Ag-Cd-Ni-Zn-Co	Rmet_6119/copF	Cu-ATPase P-type	K6V71_00045	K7A44_00045	heavy metal translocating P-type ATPase
	Rmet_6118/copG	Conserved protein involved in resistance to copper	K6V71_00050	K7A44_00050	CopG family transcriptional regulator
	Rmet_6117/copJ	Cytochrome C oxidase, cbb3-type, subunit III	K6V71_00055	K7A44_00055	cytochrome c
	Rmet_6115/copD1	copper-binding protein	K6V71_00065	K7A44_00065	copper homeostasis membrane protein CopD
	Rmet_6114/copC1	blue copper binding protein	K6V71_00070	K7A44_00070	copper homeostasis periplasmic binding protein CopC
	Rmet_6113/copB1	Copper resistance protein B precursor (ATPase)	K6V71_00075	K7A44_00075	copper resistance protein B
	Rmet_6112/copA1	multi-Cu(II) oxidase	K6V71_00080	K7A44_00080	copper resistance system multicopper oxidase
	Rmet_6111/copR1	Two-component system regulator	K6V71_00085	K7A44_00085	heavy metal response regulator transcription factor
	Rmet_6110/copS1	Sensor of two components regulator	K6V71_00090	K7A44_00090	heavy metal sensor histidine kinase
Horizontal DNA transfer related	Rmet_6126	site-specific integrase			
	Rmet_6102	tyrosine-type recombinase/integrase			
			K6V71_00095	K7A44_00095	site-specific integrase

Figure S7. Sequences related to horizontal DNA transfer at the viscinity of the *mmf* cluster.

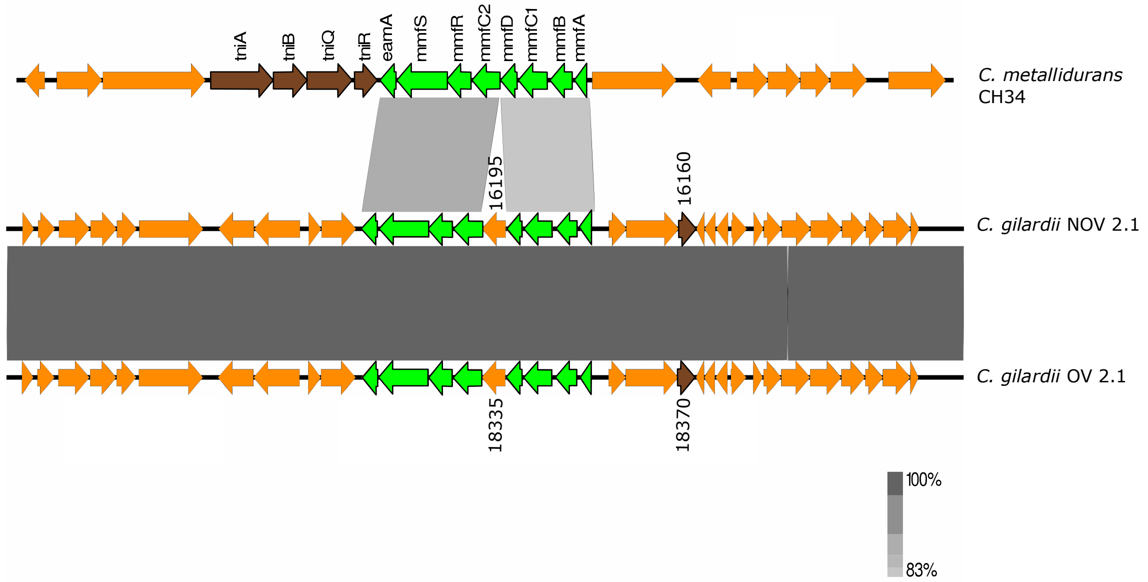

	CH34 Locus tag/Gene	Gene Anotation	Locus tag in NOV 2.1	Locus tag in OV 2.1	NCBI gene anotation
mmf system/Zn-Pb-Cd-Co	Rmet_2999/mmfA	Propeptide, PepSY amd peptidase M4 precursor (Tn6048)	K6V71_16175	K7A44_18355	PepSY domain-containing protein
	Rmet_3000/mmfB	undecaprenyl pyrophosphate phosphatase (Tn6048)	K6V71_16180	K7A44_18350	undecaprenyl-diphosphatase
	Rmet_3001/mmfC 1	putative permease MFS (Tn6048)	K6V71_16185	K7A44_18345	hypothetical protein
	Rmet_3002/mmfD	putative signal peptide protein	K6V71_16190	K7A44_18340	hypothetical protein
			K6V71_16195	K7A44_18335	DedA family protein
	Rmet_3003/mmfC 2	putative permease of the Major Facilitator Superfamily (Tn6048)	K6V71_16200	K7A44_18330	hypothetical protein
	Rmet_3004/mmfR	two component transcriptional regulator	K6V71_16205	K7A44_18325	response regulator transcription factor
	Rmet_3005/mmfS	periplasmic sensor signal transduction histidine kinase	K6V71_16210	K7A44_18320	two-component sensor histidine kinase
	Rmet_3006	putative membrane protein (Tn6048)	K6V71_16215	K7A44_18315	EamA family transporter
Horizontal DNA transfer related	Rmet_3007/tniR	serine-based site-specific recombinase activity (resolvase) (Tn6048)			
	Rmet_3008/tniQ	transposon related protein TniQ (Tn6048)			
	Rmet_3009/tniB	NTP-binding protein TniB (Tn6048)			
	Rmet_3010/tniA	Transposase (Tn6048)			
			K6V71_16160	K7A44_18370	recombinase family protein

Figure S8. Sequences related to horizontal DNA transfer at the viscinity of the *mer* cluster.

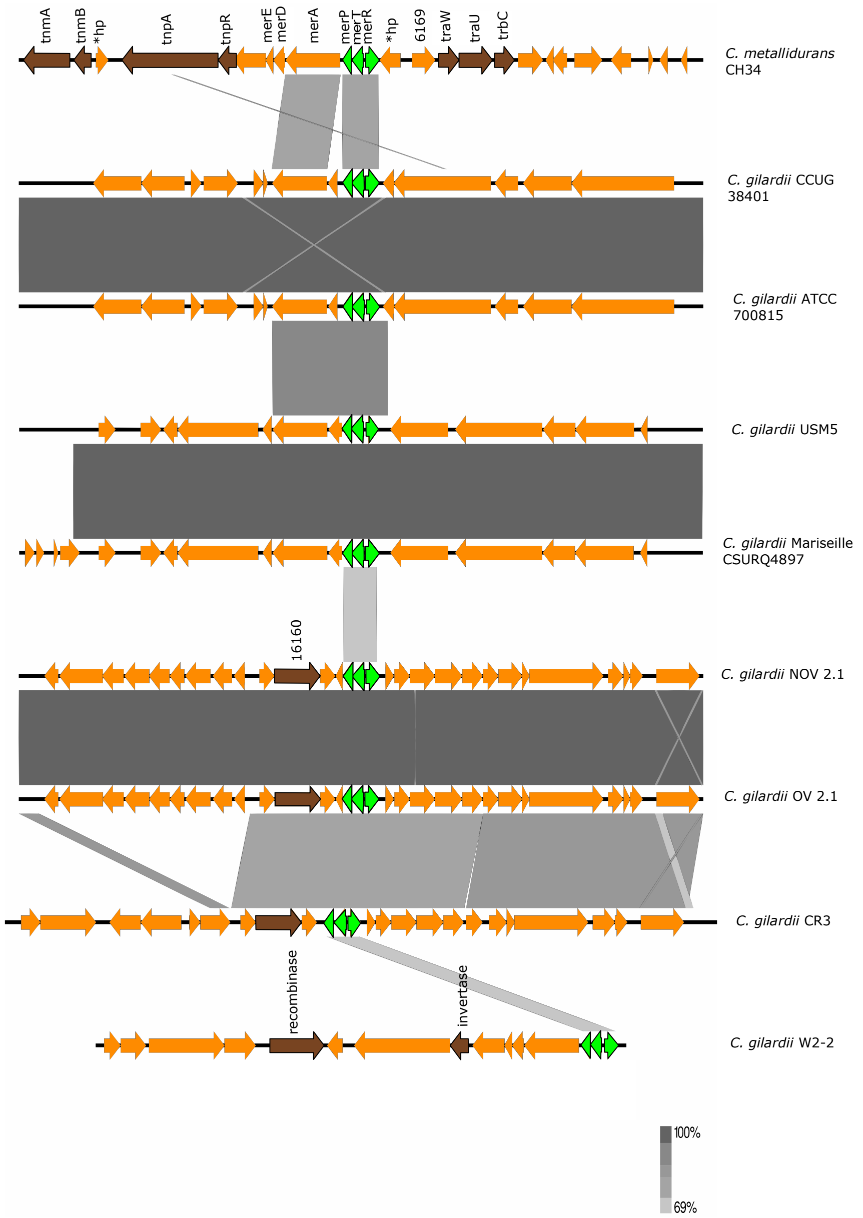

System/ Metal	CH34 Locus tag and gene (pMOL30)	Gene Anotation	Locus tag in NOV 2.1	Locus tag in OV 2.1	NCBI gene anotation
Horizontal DNA transfer related	Rmet_6182/tnmA	IS481 family transposase			
	Rmet_6181/tnmB	Transposase			
	Rmet_6179/tnpA	Tn3 family transposase			
	Rmet_6178/tnpR	Recombinase family protein			
	Rmet_6176/merE	Broad-spectrum mercury transporter MerE			
	Rmet_6175/merD	Mercury resistance co-regulator MerD			
	Rmet_6174/merA	mercury(II) reductase			
mer system/Hg-Cd-Zn	Rmet_6173/merP	Periplasmic mercury ion-binding protein	K6V71_16145	K7A44_18385	mercury resistance system periplasmic binding protein MerP
	Rmet_6172/merT	Hypothetical transport integral membrane protein	K6V71_16140	K7A44_18390	mercuric ion transporter MerT
	Rmet_6171/merR	Activator/repressor of mer operon	K6V71_16135	K7A44_18395	Hg(II)-responsive transcriptional regulator
	Rmet_6169	DsbC family protein			
Horizontal DNA transfer related	Rmet_6168/traW	Type-F conjugative transfer system protein TraW			
	Rmet_6167/traU	TraU family protein			
	Rmet_6166/trbC	Type-F conjugative transfer system pilin assembly protein TrbC			
			K6V71_16160	K7A44_18370	recombinase family protein
